# Clinical relevance and functional significance of cell-free microRNA-1260b expression profiles in infiltrative myxofibrosarcoma

**DOI:** 10.1038/s41598-020-66120-8

**Published:** 2020-06-10

**Authors:** Takuya Morita, Tomohiro Fujiwara, Aki Yoshida, Koji Uotani, Masahiro Kiyono, Suguru Yokoo, Joe Hasei, Toshiyuki Kunisada, Toshifumi Ozaki

**Affiliations:** 1 Department of Orthopaedic Surgery, Okayama University Graduate School of Medicine, Dentistry, and Pharmaceutical Sciences, Okayama, Japan; 2 Orthopaedic Service, Department of Surgery, Memorial Sloan-Kettering Cancer Center, New York, USA

**Keywords:** Sarcoma, Translational research

## Abstract

Infiltrative tumor growth into adjacent soft tissues is a major cause of the frequent recurrence and tumor-related death of myxofibrosarcoma (MFS), but no useful biomarkers reflecting tumor burden and infiltrative growth are available. While emerging evidence suggests a diagnostic and functional role of extracellular/circulating microRNA (miRNA) in various malignant diseases, their significance in MFS patients remains unknown. Global miRNA profiling identified four upregulated miRNAs in MFS patient sera and culture media of MFS cells. Among these, serum *miR-1260b* level was significantly upregulated in patient serum discriminating from healthy individuals and closely correlated with clinical status and tumor dynamics in MFS-bearing mice. In addition, high *miR-1260b* expression in serum was correlated with radiological tail-like patterns, characteristic of the infiltrative MFS. The extracellular *miR-1260b* was embedded in tumor-derived extracellular vesicles (EVs) and promoted cellular invasion of MFS through the downregulation of *PCDH9* in the adjacent normal fibroblasts. Collectively, circulating *miR-1260b* expression may represent a novel diagnostic target for tumor monitoring of this highly aggressive sarcoma. Moreover, EV-*miR-1260b* could act as a transfer messenger to adjacent cells and mediate the infiltrative growth of MFS, providing new insights into the mechanism of infiltrative nature via crosstalk between tumor cells and their microenvironment.

## Introduction

Lack of blood-based biomarkers is a major problem in the management of bone and soft tissue sarcomas (STSs). Detection of primary and/or recurrent tumors has generally relied on local symptoms and imaging modalities. Myxofibrosarcoma (MFS), one of the most common locally aggressive STSs^1,2^, is no exception. MFS comprises a spectrum of malignant fibroblastic lesions with variably myxoid stroma, pleomorphism, and a distinct curvilinear vascular pattern^3^. In addition, it is clinically characterized by infiltrative growth into surrounding soft tissues^4–6^. The infiltrative growth pattern is observed as a tail-like pattern on MRI and high correlations (87–100%) between radiological and pathological infiltration have been reported^7–9^. Since the efficacy of radiotherapy and chemotherapy are limited in MFS, surgical resection with a wide margin is standard treatment^4–6^. However, the local recurrence rate is high, ranging from 22% to 79%, even after wide resection^1,8,10,11^, and infiltrative growth is recognized as a primary risk factor for local recurrence and possibly distant metastasis^4–6,12–14^. Approximately 30% of recurrent MFS progress to higher grade tumors, which exhibit increased metastatic potential and poor prognosis^15^. However, there is no biomarker for monitoring tumor recurrence and the molecular mechanisms underlying the infiltrative nature of MFS remain unknown.

Emerging evidence suggests that microRNAs (miRNAs) play a crucial role in tumor initiation, progression, and metastasis^16–18^. miRNAs are small, non-coding RNA molecules consisting of approximately 20–22 nucleotides, which interact with the 3′ untranslated region of specific target mRNAs. These miRNAs are involved in various biological processes, including cellular development, proliferation, differentiation, and apoptosis^19,20^. Aberrant expression of a variety of miRNAs has been identified in various human malignant tumors over the past decades, which has been shown to contribute to tumor development through various mechanisms, including deletions, amplifications, and mutations involving miRNA loci, epigenetic silencing, or the dysregulation of transcription factors that target specific miRNAs^17,18^. These miRNAs can function as tumor promoters or suppressors based on their upregulated or downregulated expression levels, depending on their specific target mRNAs^21,22^.

Recent evidence has shown that tumor cells actively secrete miRNAs into the extracellular space and circulation^23,24^. Such cell-free miRNAs (cfmiRNA) exist with remarkable stability in the various types of body fluids within EVs or apoptotic bodies^25–27^. In addition, the circulating miRNAs in the bloodstream have been demonstrated to be potential novel biomarkers for patients with malignant diseases^24,28–30^. The results of several studies also suggest that circulating miRNAs can be transferred to other cells and act as mediators of cell-cell communication in a process mediated by extracellular vesicles (EVs), including exosomes; small (30–150 nm) membrane vesicles of endocytic origin that contain cellular RNA, DNA, and proteins within them^28,31,32^. The intercellular communication mediated by EVs has been shown to have a role in various cell types, including cancer cells^32^. One example includes that EVs, released by cancer cells, promote cancer migration, invasion, and metastasis, through transfer of their oncogenic, bioactive molecules such as lipids, proteins, and nucleic acids to normal cells, adjacent cancers, or specific organs^31–37^. However, it remains unclear whether circulating miRNAs are associated with tumor progression and are applicable in liquid biopsy in patients with sarcomas.

The purpose of the present study was to investigate the expression profiles of extracellular/circulating miRNAs derived from MFS cells and patients’ serum and to determine the specific miRNAs that are useful for disease monitoring. In addition, we aimed to investigate the functional significance of the extracellular miRNAs in tumor progression of MFS to understand the molecular mechanisms underlying the local aggressiveness.

## Results

### Global microRNA profiling analysis of cfmiRNA in MFS patient serum and MFS cell culture media

**Figure 1:**
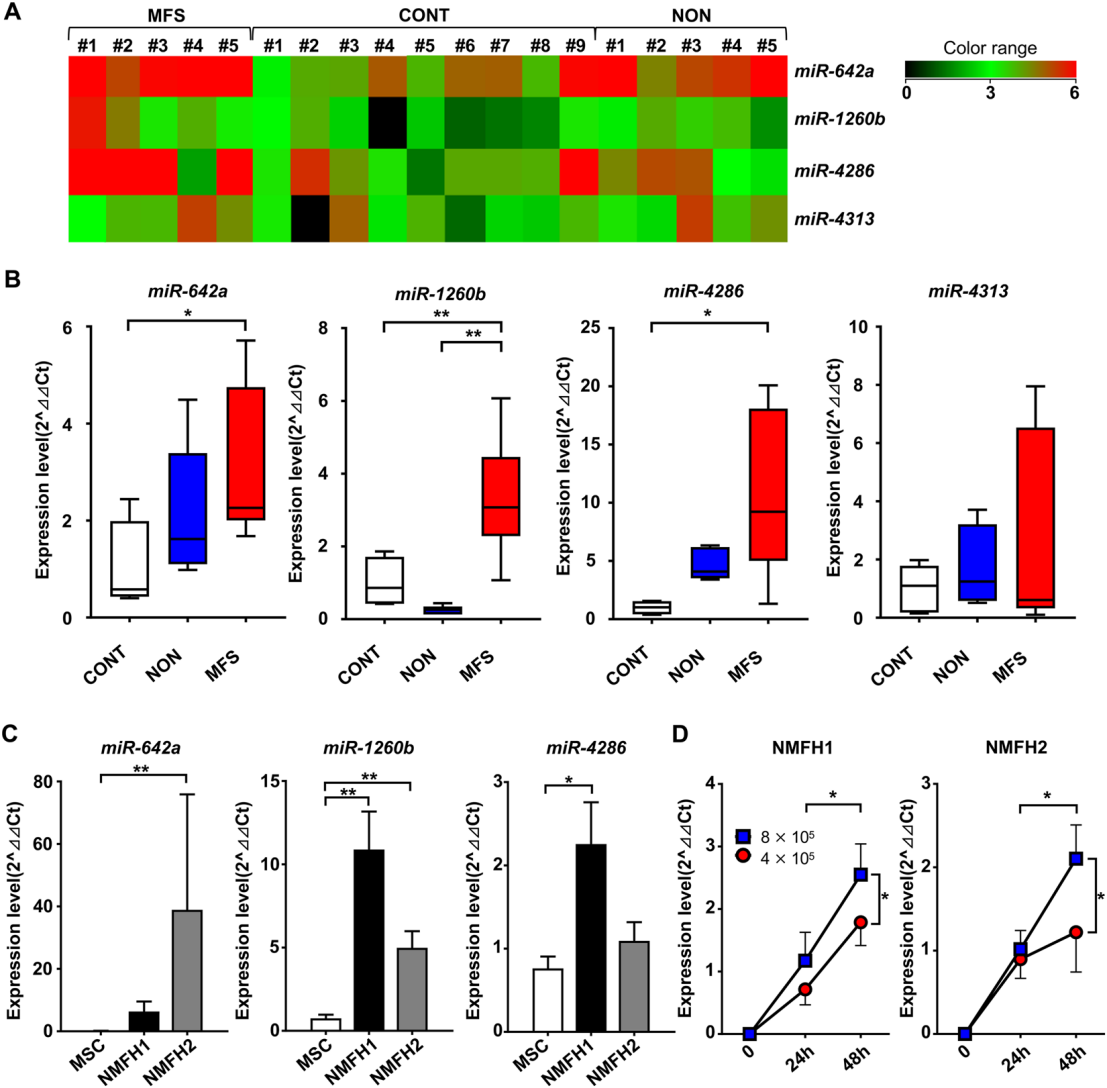
Global screening of circulating/extracellular miRNA in patients and MFS cell culture media. (**A**) Heatmap and the hierarchical clustering of miRNA microarray analysis using serum samples collected from MFS patients in preoperative state (MFS), age-matched benign tumor patients (NON), and healthy individuals (CONT). (**B**) Serum levels of four upregulated miRNAs in (**A**). *p < 0.05, **p < 0.01; Student’s *t* test. (**C**) The expression levels of extracellular *miR-642a, miR-1260b*, and *miR-4286* in MFS cell culture media (NMFH-1 and NMFH-2) compared to human mesenchymal stem cells (hMSCs). Data are presented as mean ± S.D. (n = 3 in each group) *p < 0.05, **p < 0.01; Student’s *t* test. (**D**) The expression levels of extracellular *miR-1260b* in the culture media of NMFH-1 and NMFH-2 cells based on cell number and the culture duration. Data are presented as mean ± S.D. (n = 3 in each group) *p < 0.05; Student’s *t* test.

To investigate the dysregulated expression of miRNAs in MFS patients’ serum, miRNA microarray profiling analysis was performed using serum samples and culture media of MFS cells: serum collected from MFS patients (n = 5; Supplementary Table 1), age-matched benign tumor patients (n = 5; Supplementary Table 2), healthy individuals (n = 9; Supplementary Table 3), and MFS cell culture media (n = 1; NMFH-1). Among a total of 2006 analyzed miRNAs, 110 miRNAs were significantly upregulated in the serum collected from MFS patients (*p* < 0.05) compared to the serum collected from age-matched patients with benign tumors (NON), which included 89 miRNAs expressed in the MFS cell culture media (Supplementary Table 4). On the other hand, we identified 12 miRNAs that were significantly upregulated in the MFS serum samples (*p* < 0.05) compared to the serum collected from healthy individuals (CONT), in which 11 miRNAs had their expression in the MFS cell culture media (Supplementary Table 5). Among them, four upregulated miRNAs (*miR-642a*, *miR-1260b*, *miR-4286*, *miR-4313*) were commonly upregulated in the MFS serum samples, which all had their expression in the MFS cell culture media (Fig. 1A, Supplementary Table 6).

### Upregulation of serum *miR-1260b* in MFS patients

We subsequently validated the results of microarray analysis by an independent qRT-PCR. Among four candidates, the expression levels of *miR-642a* and *miR-4286* were significantly higher in serum collected from MFS patients than healthy individuals (*p* < 0.05; Fig. 1B). In addition, only *miR-1260b* expression was significantly upregulated in the serum of MFS patients compared with healthy individuals and benign tumor patients (*p* < 0.01; Fig. 1B). The expression levels of the miRNAs were also investigated in 10 pairs of tumor and normal tissue specimens. None of the four candidates was upregulated in MFS tissue specimens compared to the normal tissue specimens (Supplementary Fig. 1).

We further analyzed the expression levels in the culture media of MFS cells and control hMSC cells. While *miR-642a* and *miR-4286* expression levels were upregulated in the culture media of either NMFH-1 or NMFH-2, *miR-1260b* levels were significantly upregulated in the culture media of both NMFH-1 and NMFH-2 cells compared to hMSC (Fig. 1C). The extracellular *miR-1260b* expression levels in MFS cell culture media increased based on culture duration and cell number (Fig. 1D). The results indicated that *miR-1260b* was secreted into the extracellular spaces from the MFS cells. Therefore, we focused on this miRNA in further investigations.

### Clinical relevance of serum *miR-1260b* levels in MFS patients

**Figure 2:**
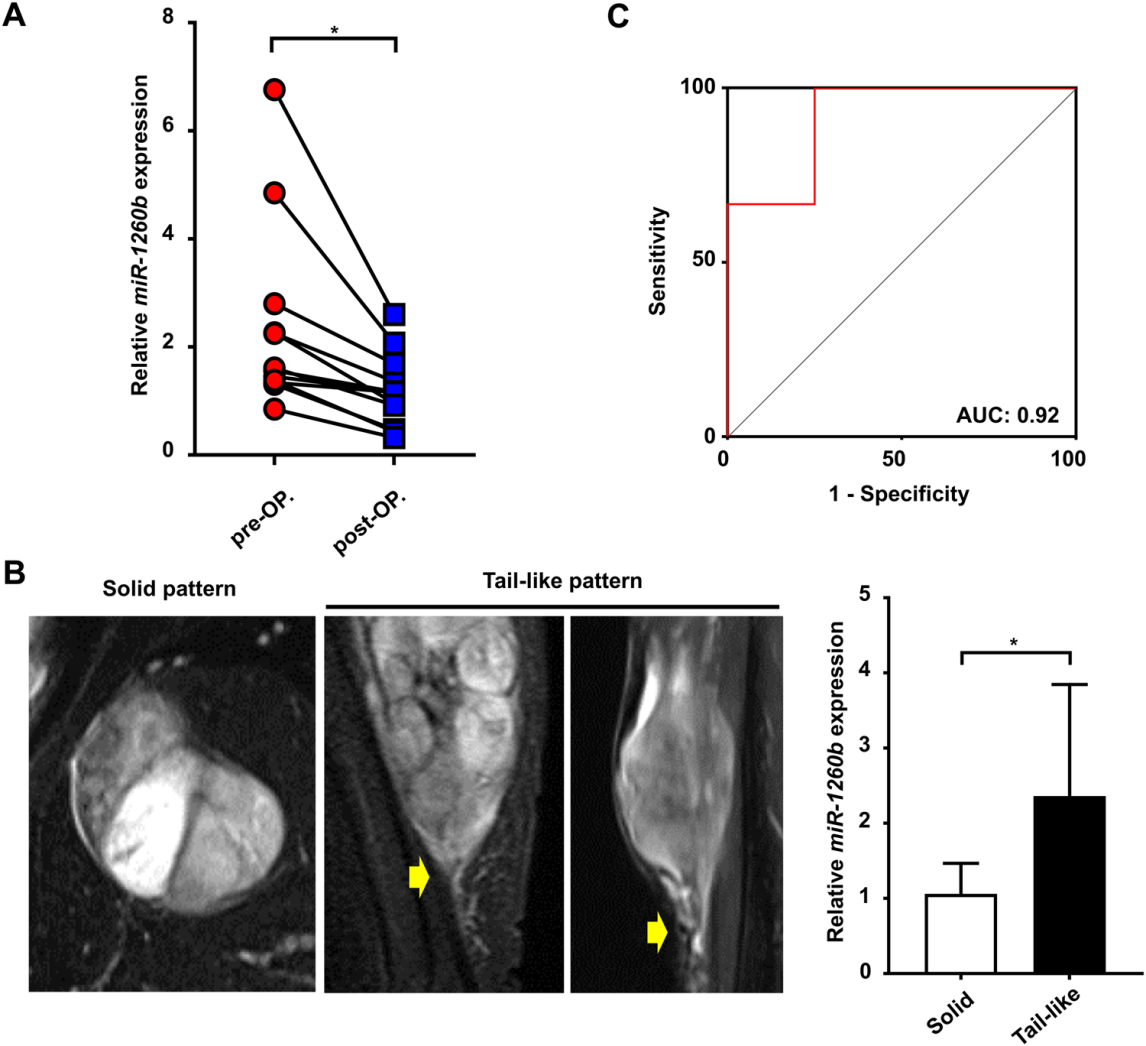
Clinicopathological relevance of serum *miR-1260b* in MFS patients. (**A**) Serum *miR-1260b* expression levels in pre- and postoperative states. *p < 0.05; Wilcox signed-rank test. (**B**) Gd-T1WI MR imaging patterns of MFS. Left, solid pattern; center, tail-like pattern; right, serum *miR-1260b* levels in patients with/without tail-like pattern (n = 4, solid pattern; n = 6, tail-like pattern). *p < 0.05; Mann-Whitney *U*-test. (**C**) Receiver operating characteristic (ROC) curve analysis. ROC curve analysis indicated an AUC of 0.92 (95% confidence interval; 0.73–1.11) distinguishing patients with solid pattern from those with tail-like pattern.

We further investigated the clinical relevance of serum *miR-1260b* levels to determine whether circulating miRNA could be used to monitor tumor dynamics in MFS patients. The serum samples collected at pre- and postoperative states from 10 MFS patients who underwent tumor wide resection were analyzed (Table 1). Eight patients were available for the administration of a pair of serum at the pre- and postoperative states. The serum *miR-1260b* levels significantly decreased postoperatively in all patients (*p* = 0.039; Fig. 2A). Subsequently, we compared the serum *miR-1260b* levels in patients with or without the tail-like pattern, which implies infiltrative growth of the disease^7–9^. Notably, serum *miR-1260b* expression levels at diagnosis in the sera from patients with the tail-like pattern (n = 6) were significantly higher than in those collected from patients without the tail-like pattern (solid pattern; n = 4; *p* = 0.038; Fig. 2B, Table 2). A receiver operating characteristic (ROC) curve was then generated to evaluate the sensitivity and specificity of serum *miR-1260b* as a biomarker for the infiltrative pattern in MFS patients. Circulating *miR-1260b* discriminated MFS with the tail-like pattern from MFS with the solid pattern with an area under the curve (AUC) value of 0.92 (95% confidence interval = 0.7281 to 1.1050; Fig. 2C). The sensitivity and specificity values for identifying MFS patients with the tail-like pattern were 83.3% and 75%, respectively. The results demonstrated that serum *miR-1260b* expression levels are associated with not only tumor burdens but also the infiltrative nature of MFS, indicating the usefulness of serum *miR-1260b* as a circulating biomarker for disease monitoring and the functional relevance of circulating *miR-1260b* in the infiltrative growth pattern of MFS.

**Table 1:** Clinical characteristics of patients with myxofibrosarcoma.

No.	Gender	Age at diagnosis	Site	Depth	MRI pattern	Size (cm^3^)	Histological grade (FNCLCC)	Local recurrence	Distant metastasis	Oncologic outcome
1	Male	65	Trunk	Superficial	Tail-like	19.7	2	−	−	CDF
2	Male	83	Extremity	Superficial	Solid	3.5	2	−	−	CDF
3	Male	83	Trunk	Deep	Tail-like	11.6	2	−	−	CDF
4	Female	87	Extremity	Deep	Tail-like	131.0	1	−	+	AWD
5	Male	80	Extremity	Deep	Solid	34.3	2	−	−	CDF
6	Male	65	Extremity	Deep	Tail-like	225.0	2	−	−	CDF
7	Male	88	Extremity	Deep	Tail-like	6.3	2	+	−	AWD
8	Male	63	Extremity	Deep	Tail-like	59.7	2	+	−	AWD
9	Female	77	Extremity	Deep	Solid	72.3	3	−	−	CDF
10	Female	88	Trunk	Deep	Solid	68.6	2	−	−	CDF

**Table 2:** Clinical relevance of serum *miR-1260b* expression levels.

Variable	Number of patients	Serum *miR-1260b* levels	*p* value
		(± mean SD)	
Age at diagnosis			0.476
≤80 years	6	1.22 ± 0.24 (n = 6)	
>80 years	4	2.26 ± 1.46 (n = 4)	
Gender			0.383
Male	7	1.91 ± 1.24 (n = 7)	
Female	3	1.69 ± 1.27 (n = 3)	
Tumor size			0.714
≤5.0 cm	3	1.59 ± 0.35 (n = 6)	
>5.0 cm	7	1.95 ± 1.46 (n = 4)	
MRI pattern			0.038
Tail-like pattern	6	2.36 ± 1.36 (n = 6)	
Solid pattern	4	1.06 ± 0.36 (n = 4)	
Local recurrence			0.651
No	8	1.58 ± 0.43 (n = 8)	
Yes	2	1.91 ± 1.37 (n = 2)	

### Extracellular *miR-1260b* embedded in the tumor-derived EVs

**Figure 3:**
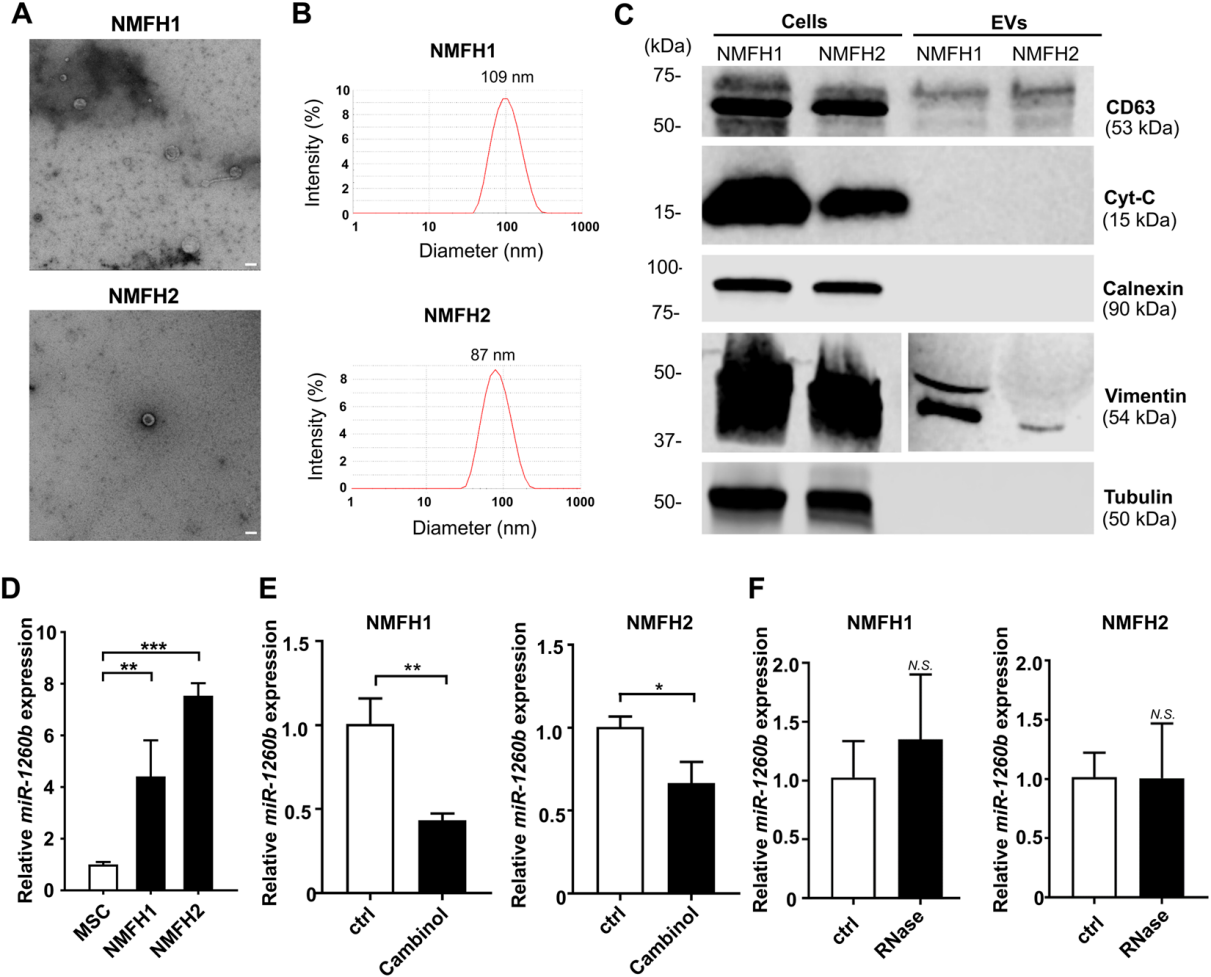
Extracellular *miR-1260b* embedded on MFS-derived EVs. (**A**) Negative stain transmission electron microscopy (TEM) image of MFS cell lines (NMFH-1 and NMFH-2)-derived EVs. The image shows small vesicles approximately 100–150 nm in diameter. Scale bar =100 nm. (**B**) Representative dynamic light scattering analysis of EVs obtained from NMFH-1 and NMFH-2. The graph reports the diameter (nm) of the particles (x-axis) versus intensity (Optical Density y-axis). Numbers on graphs are the EV average sizes of NMFH-1 and NMFH-2. The isolated EVs ranged in size peak of 109 nm and 87 nm. (**C**) Western blot analysis on purified EVs derived from NMFH-1 and NMFH-2 and parental cellular lysates. CD63, a conventional EV marker. Cytochrome-C (Cyt-C), a mitochondrial marker. Calnexin, an endoplasmic reticulum (ER) marker. Vimentin, a mesenchymal marker. Tubulin as internal control for Western blot analysis. Full-length blots are presented in Supplementary Fig. 3. (**D**) EV-*miR-1260b* levels normalized to *cel-miR-39* in MFS cells and hMSCs. Data are mean ± S.D. (n = 3 in each group) **p < 0.01, ***p < 0.001; Student’s t test. (**E**) *EV-miR-1260b* levels normalized to *cel-miR-39* in MFS cells treated with the presence of absence of cambinol (48 hours). Data are mean ± S.D. (n = 3 in each group). *p < 0.05, **p < 0.01; Student’s *t* test. (**F**) *EV-miR-1260b* levels normalized *t*o *cel-miR-39* in MFS cells treated with the presence of absence of RNase. Data are mean ± S.D. (n = 3 in each group). *N.S*., not significant; Student’s *t* test.

In order to understand the intracellular and extracellular dynamics of *miR-1260b* in MFS cells, we investigated the intracellular expression level of *miR-1260b* in the tumor-derived EVs secreted by the MFS cells. The intracellular expression levels of *miR-1260b* were not upregulated in the MFS cells compared to those in the control hMSC cells (Supplementary Fig. 2). Next, we characterized the purified EVs from the culture media of both NMFH-1 and NMFH-2 cells. Negative-stain transmission electron microscopy (TEM) revealed that the isolated particles were essentially homogeneous vesicles approximately 100 to 150 nm in diameter (Fig. 3A). Dynamic light-scattering analysis using Zetasizer Nano-S confirmed the presence of vesicles with a peak size of 109 nm and 87 nm in NMFH-1 and NMFH-2 cells, respectively (Fig. 3B). Western blotting of the EV fractions confirmed the expression of the tetraspanin protein, CD63, which is a known EV marker. However, only whole-cell lysates showed the expression of cytochrome-C (Cyt-C), a mitochondrial marker, and calnexin, an endoplasmic reticulum (ER) marker, supporting the quality of EVs preparations (Fig. 3C, Supplementary Fig. 3). In contrast, the whole-cell lysates and EVs showed the expression of vimentin, a mesenchymal marker. Additionally, the whole-cell lysates showed the expression of tubulin (Fig. 3C, Supplementary Fig. 3). We then investigated the expression level of *miR-1260b* in the EV fractions and observed significant elevated expression compared with the EVs purified from control hMSCs (Fig. 3D). We also checked the expression level of *miR-1260b* after depletion of EVs from MFS cells using cambinol, an inhibitor of neutral sphingomyelinase-2, which plays a regulatory function in EVs budding from the plasma membrane^38,39^. Cambinol treatment significantly decreased extracellular *miR-1260* levels in both NMFH-1 and NMFH-2 cells (Fig. 3E). In addition, we treated secreted EVs with RNase before qRT-PCR to confirm that miR-1260b is embedded in EVs. No significant differences in the RNA amount and *miR-1260b* and *cel-miR-39* expression levels were observed between the RNase A treated group and the control group (Fig. 3F). The results indicated that the extracellular *miR-1260b* was embedded in the MFS-derived EVs. Furthermore, we isolated EVs from serum samples obtained from MFS patients and healthy individuals in order to investigate *miR-1260b* expression levels from the EVs. *miR-1260b* expression in the patient-derived EVs was also amplified, and was found to be significantly upregulated in the EVs from MFS patients compared with healthy individuals (Supplementary Fig. 4).

### Enhanced infiltration of MFS by EV-*miR-1260b* via stimulation of normal fibroblasts (NFs)

**Figure 4:**
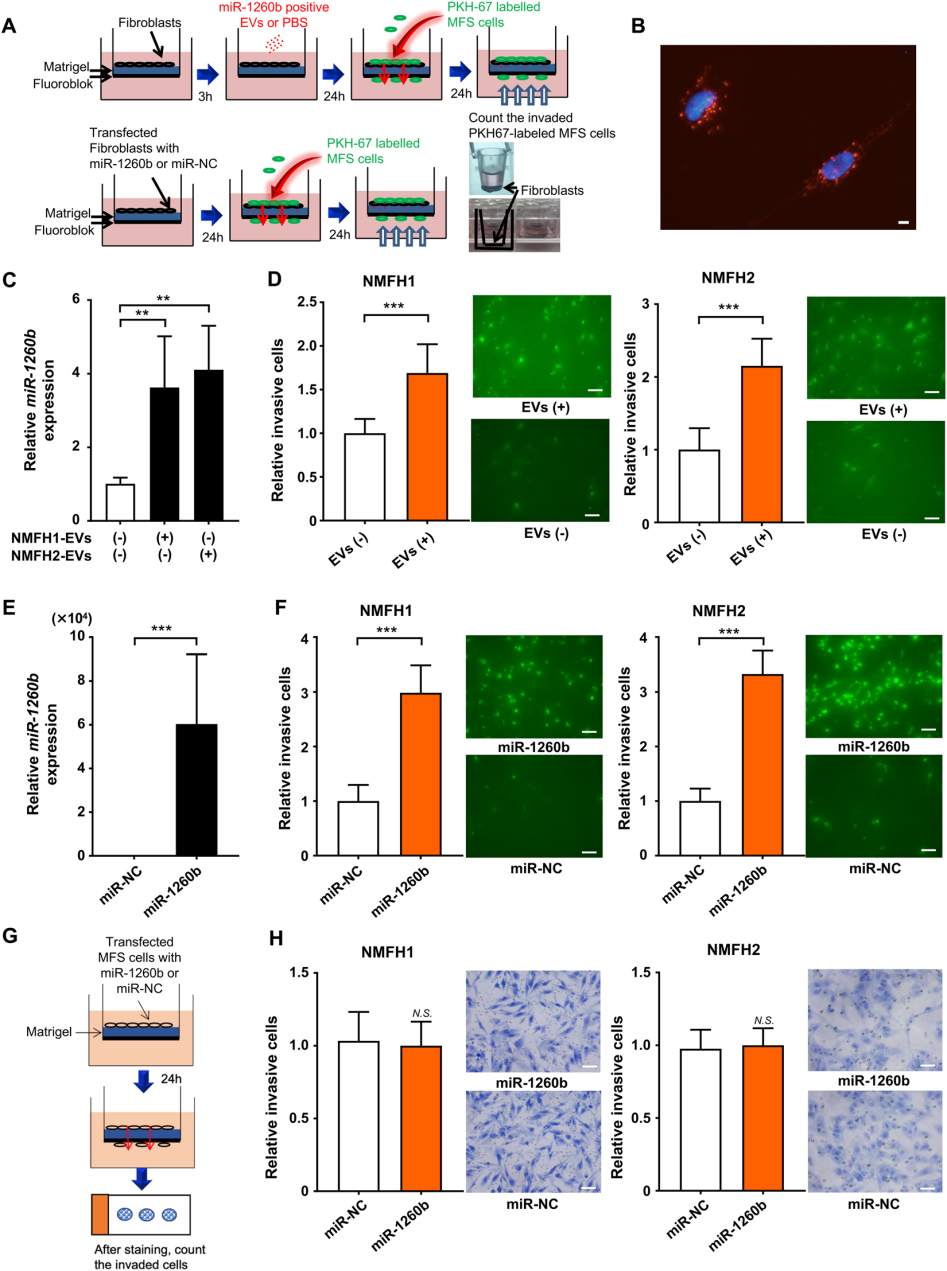
Tumor infiltration promoted by crosstalk between MFS cells and NFs. (**A**) Schema of invasion assay using MFS cells and normal fibroblasts educated by tumor-derived EVs or synthetic *miR-1260b*/control. (**B**) Fluorescence microscope shows PKH-67-labeled EVs derived from MFS were captured in NFs. Scale bar = 100 nm. (**C**) Upregulated *miR-1260b* levels in NFs educated using MFS-derived EVs. **p < 0.01; Student’s *t* test. (**D**) Invasion assay. Left, NMFH-1; right, NMFH-2. EV-educated NFs derived from MFS cells promoted cellular invasion of MFS cells. ***p < 0.001; Student’s *t* test. (**E**) Upregulated *miR-1260b* levels in NFs transfected with synthetic *miR-1260b* mimic or control. ***p < 0.001; Student’s *t* test. (**F**) Invasion assay. Left, NMFH-1; right, NMFH-2. NFs upregulated with *miR-1260b* mimic promoted cellular invasion of MFS cells. ***p < 0.001; Student’s *t* test. (**G**) Schema of invasion assay using MFS cells transfected with synthetic *miR-1260b* or control. (**H**) Invasion assay. Left, NMFH-1; right, NMFH-2. Scale bar = 100 μm. *N.S*., not significant; Student’s *t* test.

To further investigate the functional significance of extracellular *miR-1260b*, we performed an invasion assay using MFS cells and NFs, educated using tumor-derived EVs and synthetic *miR-1260b*. We established an *in vitro* model mimicking the tumor microenvironment using MFS cells, NFs, and matrigel chamber. NFs, which had been transfected with synthetic *miR-1260b* or educated using tumor-derived EVs, were plated onto growth factor reduced matrigel (Fig. 4A). We first labeled MFS-derived EVs with PKH67 and cultured them with NFs, which enabled us to detect tumor-derived EVs taken up by NFs (Fig. 4B). We then analyzed the expression levels of *miR-1260b* in NFs educated using NMFH1- and NMFH2-derived EVs. *miR-1260b* expression levels were significantly higher in the EV-educated control NFs (Fig. 4C). In addition, the education of the EVs on NFs significantly stimulated the invasion of MFS cells (Fig. 4D). We further assessed the cellular invasiveness of MFS using *miR-1260b* overexpressed NFs. First, we confirmed that *miR-1260b* were overexpressed in the NFs with synthetic *miR-1260b* transfection compared to miR-NC (Fig. 4E). We then observed that cellular invasiveness in NMFH-1 and NMFH-2 was enhanced in the co-culture environment with *miR-1260b* overexpressed NFs (Fig. 4F). The results suggested that EV- *miR-1260b* promoted cellular invasion of MFS via the stimulation of NFs. Finally, we assessed the cellular invasiveness of *miR-1260b* overexpressed MFS without NFs (Fig. 4G). The numbers of invaded NMFH-1 and NMFH-2 cells, overexpressing *miR-1260b*, were not enhanced without in the absence of NFs (Fig. 4H). We also evaluated the effect of EVs from NFs in MFS cells in terms of cellular invasion. The cellular invasion of MFS cells did not change with or without education of EVs from NFs, indicating the EVs from NFs have minimal effect on MFS invasion (Supplementary Fig. 5). Collectively, the results above demonstrate that EV-*miR-1260b* induces MFS cell invasion not through “autocrine” but through “paracrine” mechanisms from MFS cells, communicating with the adjacent normal mesenchymal cells.

### *PCDH9* as a direct target of *miR-1260b* in fibroblasts regulating MFS infiltration

**Figure 5:**
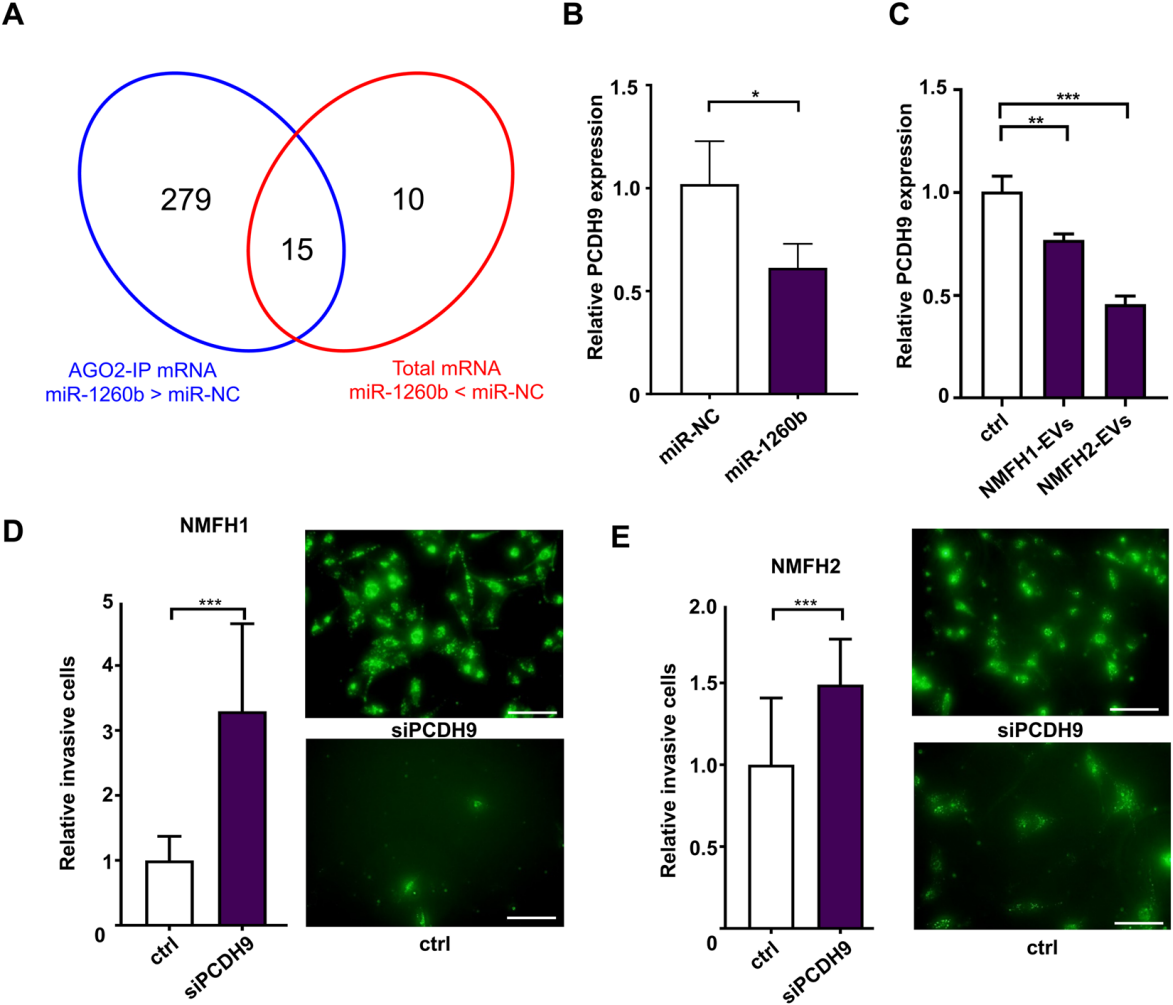
Downregulation of PCDH9 by *miR-1260b* in NFs promotes MFS cell invasion. (**A**) Venn diagram of mRNA microarray analysis using RNA collected from AGO2-IP and cellular transfection with synthetic *miR-1260b* and *miR-NC*. (**B**) The expression levels of PCDH9 in NFs transfected with *miR-1260b* or *miR-NC*. *p < 0.05. (**C**) The expression levels of PCDH9 in NFs treated with EVs secreted from NMFH-1 and NMFH-2. **p < 0.01. (**D,E**) Invasion assay using NFs transfected with siPCDH9 and negative control siRNA (ctrl). Twenty-four hours later, the invaded PKH67-labeled NMFH-1 cells (**D**)NMFH-2 cells (**E**) were counted. Data B–E are mean ± S.D. (n = 3 in each group) **p < 0.01; ***p < 0.001; Student’s *t* test.

To identify target genes of *miR-1260b*, we carried out mRNA profiling using two different microarray analyses. Twenty-five genes were identified as decreased mRNAs with at least two-fold changes in the *miR-1260b* overexpressed NFs compared to the control NFs (miR-NC; Supplementary Table 7). The second microarray was performed using RNA collected using anti-Argonaute 2 antibody immunoprecipitation (Ago-2 IP) in NF cells transfected with *miR-1260b* or miR-NC. A total of 294 genes were identified as upregulated mRNAs with at least four-fold changes in the former samples (Supplementary Table 8). The combined data identified 15 candidate genes as *miR-1260b* targets (Fig. 5A, Supplementary Table 9). Among the 15 candidate genes, qRT-PCR revealed that *PCDH9* was significantly downregulated in NFs with *miR-1260b* overexpression (Fig. 5B) or treatment with EVs derived from MFS cells (Fig. 5C). Finally, we investigated whether the targets were correlated with cellular invasiveness of MFS through the stimulation of NFs. Silencing of *PCDH9* in the NFs promoted cellular invasion of MFS in the co-culture environment of MFS cells and NFs (Fig. 5D,E). The results indicated that tumor-derived EV-*miR-1260b* promote local aggressiveness of MFS via the downregulation of *PCDH9* in the peri-tumoral normal mesenchymal cells.

### *In vivo* dynamics of serum *miR-1260b* expression levels in MFS-bearing mice

**Figure 6:**
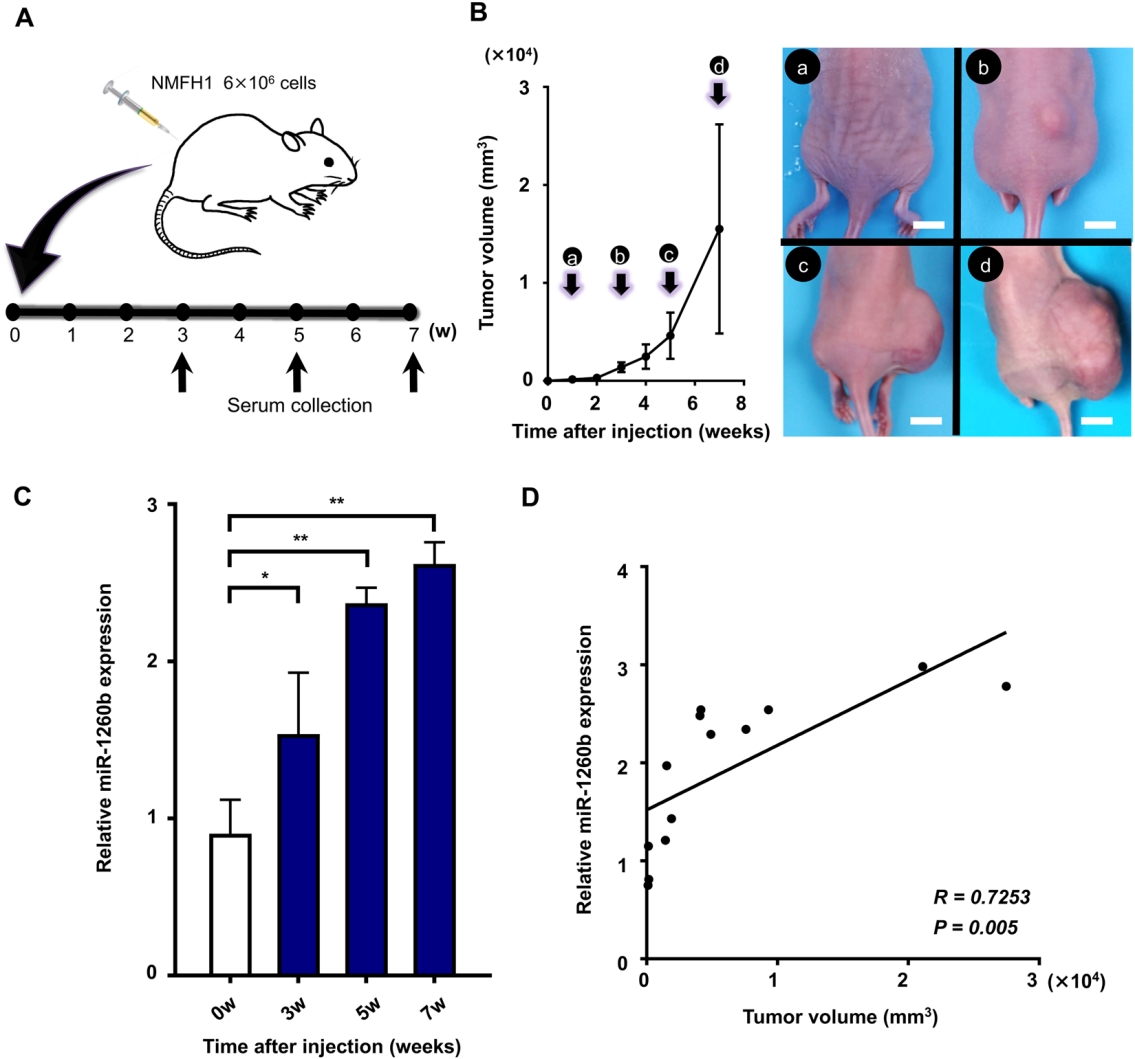
Serum *miR-1260b* levels in MFS-bearing mice. (**A**) Schema of the animal experiments. (**B**) Tumor volumes were measured each week after MFS cell injection (left). The image shows tumor growth at one (a), three (b), five (c) and seven (d) weeks after inoculation (right). Scale bar = 10 mm. (**C**) Serum *miR-1260b* levels at indicated time points. *p < 0.05, **p < 0.01; Student’s *t* test. (**D**) Pearson’s correlation between serum *miR-1260b* expression levels and tumor volume (R = 0.7253, *p* = 0.005).

Finally, we established an MFS xenograft mouse model using the NMFH-1 cell line and investigated the correlations between tumor growth and serum *miR-1260b* levels in order to evaluate the *in vivo* dynamics of serum *miR-1260b* expression levels (Fig. 6A). Along with increased tumor size (Fig. 6B), serum *miR-1260b* expression levels were elevated significantly in MFS-bearing mice (Fig. 6C). In addition, we observed a significant correlation between tumor volume and serum *miR-1260b* expression levels (*R* = 0.7253, *p* = 0.005; Fig. 6D). These results, together with the results using patients’ serum (Fig. 1B, Fig. 2A–C), suggested that serum expression of *miR-1260b* could reflect tumor burden in MFS-bearing animals and humans.

## Discussion

Emerging evidence has demonstrated the potential clinical utility of circulating miRNAs as diagnostic and prognostic biomarkers in malignant diseases. Several studies have demonstrated the potential in various types of cancers including lung^40–42^, breast^43–45^, prostate^46,47^, colorectal^48,49^, gastric^50–52^ and liver cancers^53–55^. However, the evidence for STSs is limited to synovial sarcoma^30,56^, rhabdomyosarcoma^57^, and MPNST^58^. In addition, the functional relevance of circulating miRNAs with tumor progression is rarely discussed in sarcomas. To the best our knowledge, the present study is the first to describe the global profiling of circulating miRNAs in patients with MFS and to demonstrate their functional role in local MFS aggressiveness.

We observed dissimilar miRNA dysregulation patterns between intracellular and extracellular miRNA expression. For example, three miRNAs (*miR-642a, miR-1260b*, and *miR-4286*) were upregulated in MFS cell culture media compared to control hMSCs, while the cellular expression of the miRNAs was not upregulated in the MFS cells compared to the control hMSC cells. The dissimilarity between cellular miRNAs and secreted miRNAs are also observed in various cancers, such as breast cancer^59–61^, osteosarcoma^29^, and synovial sarcoma^30^. Although the reason for the dissimilarity remains unknown, the existence of molecular mechanisms regulating the secretion of miRNAs might exist. Therefore, our global miRNA profiling approach focusing on the extracellular/circulating miRNAs is potentially appropriate for the investigation of clinically useful miRNA candidates rather than focusing on intracellular miRNA dysregulation of malignant tumors. Although we did not identify any differences in *miR-1260b* expression between tumor cells/tissues and normal cells/tissues, differences were observed when we focused on circulating miRNAs, which supports their use as sources to look for new biomarkers for diagnosis or disease monitoring in a noninvasive way.

To date, the oncogenic role of *miR-1260b* has been reported in prostate cancer, renal cell carcinoma, non-small cell lung cancer, and colorectal cancer^62–65^. Silencing of *miR-1260b* in prostate cancer cells enhanced the expression of *sFRP1* and *Smad4* and decreased cellular proliferation, invasion, and migration^62^. In addition, *miR-1260b*, highly expressed in renal cell carcinoma, promoted cellular proliferation and invasion via the reduction of the expression of several tumor suppressor genes associated with Wnt signaling, such as *sFRP1*, *Smad4*, and *Dkk2*^63^. The overexpression of *miR-1260b* is reportedly correlated with the development of non-small cell lung cancer and prognosis of lymph node metastasis^64^. The studies above have demonstrated overexpression of intracellular *miR-1260b*, which indicate its oncogenic role in several cancer cells. However, the significance of extracellular/circulating *miR-1260b* in malignant tumors remained unclear. Therefore, our investigation is the first to describe the significance of the extracellular/circulating *miR-1260b*, which could be a promising non-invasive biomarker for the monitoring of MFS.

Recent investigations have demonstrated that extracellular miRNAs stimulate tumor progression in the tumor microenvironment^37,66–68^. Tumor-derived EVs, containing nucleic acids or proteins, have been shown to play a crucial role in signal exchange with recipient cells^69–72^. For example, extracellular *miR-150*, secreted from monocytes, could enter human vascular endothelial cells and enhance cell migration of the recipient cells^67^. In another study, extracellular *miR-9*, derived from various tumor cells, were delivered to endothelial cells and mediated breast cancer cell invasion^73^. The breast cancer-derived *miR-105* effectively reduced the expression of the junctional proteins, including ZO-1 in endothelial cells, and facilitated metastasis^37^. In the present study, extracellular *miR-1260b*, packaged in tumor-derived EVs facilitated cellular invasion of MFS cells, which was not through an “autocrine” but through “paracrine” mechanism by alternating miRNA and mRNA expression within the peri-tumoral fibroblast cells. The results indicate that tumor-derived EVs containing miRNAs of donor cells could promote the migration, invasion, and metastasis of tumor cells through intracellular communication between tumor cells and normal mesenchymal cells in the peri-tumoral microenvironment.

The infiltrative growth of MFS, which is radiologically observed as a tail-like pattern under MRI, is associated with high local recurrence rates and subsequent decreased mortality^8^. In the present study, we revealed that *miR-1260b-PCDH9* in the NFs are associated with cellular infiltration of MFS. Protocadherins (PCDHs) are a group of adhesion proteins that play an important role as regulators of cell-cell adhesion. *PCDH9* is located at 13q21.32 chromosomal loci in humans and its dysregulated expression is associated with the malignant tumor phenotype^74^. *PCDH9* is downregulated in glioblastoma and overexpression of *PCDH9* inhibits cellular invasion of glioma^75,76^. Silencing of *PCDH9* decreased the expression of epithelial markers, such as E-cadherin and Occludin^77^. The infiltrative nature of MFS could be explained by the inhibition of cell-cell adhesion between tumor and normal mesenchymal cells, which may be caused by *PCDH9* suppression via tumor-derived EV-*miR-1260b*. In this *in vitro* model, the increased invasiveness of MFS cells could be related to not only pretreatment of EV-*miR-1260* to NFs, which downregulated *PCDH9* expression, but also excessive secretion of *miR-1260b* in the extracellular space from MFS cells affecting the NFs locally.

Another unique strength of our study is that we could establish an *in vivo* animal model for liquid biopsy and validate our results from human clinical samples with the use of our model. Although our methods of MFS xenograft model may not represent the complexity in patients, serum expression levels of *miR-1260b*, selected from the array analysis using serum samples from patients and culture media of MFS cells, was significantly correlated with tumor growth. A recent report from Hur *et al*. describes an *in vivo* metastasis animal model for colorectal cancer (CRC). In their model, serum *miR-203* expression levels were significantly elevated in CRC-bearing mice with liver metastasis compared with nonmetastatic mice^78^. In addition, they reported a significantly positive correlation between serum *miR-203* levels and tumor volume of metastatic nodules^78^. Although future studies are necessary to clarify whether serum *miR-1260b* levels could also be used as a tumor-monitoring marker for metastasis of MFS, we believe that our *in vivo* animal model contributes to the establishment of liquid biopsy in other sarcoma models.

In summary, our global miRNA profiling analysis revealed the upregulation of circulating/extracellular *miR-1260b* in patients with MFS. In addition, serum *miR-1260b* expression levels were correlated with tumor burden in both MFS-bearing mice and patients and radiological tail-like sign, which represent the infiltrative nature of the tumor. Furthermore, tumor infiltration was mediated by tumor-derived EV-*miR-1260b*, which decreased *PCDH9* expression in peri-tumoral NFs cells. The above findings offer novel insights into the infiltrative nature of MFS based on cell-cell communication via tumor-derived EVs and demonstrate the potential of non-invasive blood-based monitoring in patients with the aggressive STS.

## Materials and Methods

### Patients and samples

The study protocol was approved by the Institutional Review Board. Written informed consent was obtained from all patients and healthy individuals involved in this study. Blood samples were collected from MFS patients (n = 15**;** Supplementary Table 1), age-matched nonsarcoma patients (n = 5**;** Supplementary Table 2), and healthy control individuals (n = 9**;** Supplementary Table 3) at our institute between 2013 and 2016. Among the samples collected from the 15 MFS patients, a total of five samples from five patients were used for microarray analyses and other samples (10 preoprerative and 10 postoperative) from 10 patients were used for tumor monitoring by the specific serum miRNA. Clinical data, including MRI patterns, age at surgery, gender, tumor site, tumor depth, tumor size, histological grade, presence or absence of recurrence, and presence or absence of metastasis were retrieved from the sarcoma database. Gadolinium-enhanced MRI (Gd-MRI) images were evaluated for signal characteristics and a tumor-infiltrative growth pattern. MFS exhibits an extensive multidirectional spread pattern, particularly along the fascial plane and this growth pattern is defined as a tail-like pattern^9,79^. In contrast, Gd-MRI images of some MFS cases do not exhibit extensive signal spread and good demarcation, and are described as a solid pattern^3^. Venous blood samples were preserved at 4 °C after collection from patients. Serum samples were separated from venous blood samples by centrifugation at 3,500 rpm for 15 min at 4 °C. The serum samples were then centrifuged at 20,000 × g for 15 min 4 °C to prevent contamination by cellular nucleic acids and then passed through a 0.22-µm pore filter (Merck Millipore, Billerica, MA., USA). The sera samples were stored at −80 °C until further processing.

### Cell lines and cell culture

The human MFS cell lines, NMFH-1 and NMFH-2, were obtained from Niigata University and human mesenchymal stem cells (hMSCs) were obtained from Lonza (Walkersville, USA). Both the MFS cell lines were maintained in Roswell Park Memorial Institute media (RPMI, Gibco Laboratories, Grand Island, NY, USA.). Normal skeletal muscle tissues were obtained from 3 patients undergoing wide resection of MFS tumor. Normal skeletal muscle tissues placed and cultured on collagen coating dish in culture media which select for fibroblast growth Dulbecco’s Modified Eagle’s medium (DMEM) (NACALAI TESQUE, Inc., Kyoto, Japan) for 1 week, fibroblast cells were grown. Primary cultures of normal fibroblasts (NFs) were established.

All culture media were supplemented with 10% fetal bovine serum (FBS, Hyclone, Victoria, Australia), penicillin (100 U/mL), and streptomycin (100 mg/mL) (NACALAI TESQUE) in a humidified atmosphere containing 5% CO_2_ at 37 °C.

### miRNA microarray analysis

The miRNA microarrays were manufactured by Agilent Technologies (Tokyo, Japan), and 2 ng of extracted RNA were used for each microarray experiment. RNA quantity and quality were determined using a NanoDrop ND-1000 spectrophotometer (Thermo Fisher Scientific, Massachusetts, USA) and an Agilent Bioanalyzer (Agilent Technologies, Santa Clara, CA, USA), as previously described^80^. Total RNA was labeled with cyanine 3 (Cy3) using a miRNA Complete Labeling and Hyb Kit (Agilent Technologies) following the manufacturer’s instructions. In brief, total RNA was dephosphorylated using Calf Intestinal Alkaline Phosphatase (CIP) Master Mix incubated at 37 °C for 30 min. Dephosphorylated RNA was denatured with DMSO incubated at 100 °C for 5 min and then immediately transferred to ice for 2 min. These products were mixed with a ligation master mix for T4 RNA ligase and Cy3-pCp (cyanine 3-cytidine biphosphate) and incubated at 16 °C for 2 hours. Labeled RNA was dried using a vacuum concentrator at 55 °C for 1.5 hours. Cy3-pCp-labeled RNA was hybridized on an Agilent SurePrint G3 Human miRNA 8 × 60 K Rel.19 (design ID: 046064) array at 55 °C for 20 hours. After washing, microarrays were scanned using an Agilent DNA microarray scanner. Intensity values of each scanned feature were quantified using Agilent Feature Extraction software ver. 10.7.3.1, which performs background subtractions. We only used features that were flagged as no errors (detected flags) and excluded features that were not positive, not significant, not uniform, not above background, saturated, and population outliers (not detected flags). The expression analysis was performed with Agilent GeneSpring GX software version 13.1. There are a total of 2,006 miRNA probes on SurePrint G3 Human miRNA 8 × 60 K Rel.19 (design ID: 046064) without control probes.

### RNA extraction

Total RNA of serum sample or culture media was isolated from 200 μl of serum or culture media using the miRNeasy Mini Kit (Qiagen, Valencia, CA, USA) according to manufacturer’s instructions.

### Isolation of EVs from cell culture media

EVs were isolated from culture medium (CM) supernatant of MFS cells, hMSCs, and NFs. CM from cell lines that were grown to 50–70% confluence was exchanged with FBS free culture medium and was collected after a 24-h culture. The collected CM was first centrifuged at 3,500 rpm for 15 min and 9,000 × g for 30 min at 4 °C followed by filtration through a 0.22-μm filter (Merck Millipore) to remove cellular debris, unwanted proteins, and any live or dead cells. The supernatant was concentrated to about 1 ml using 100-kDa MWCO ultrafiltration membranes (Fisher Scientific, Loughborough, UK). Afterwards, the concentrated sample was ultracentrifuged at 100,000 g for 70 min at 4 °C using Optima TL-100 (Beckman Coulter, Fullerton, CA, USA) at the Central Research Laboratory, Okayama University Medical School. The supernatant was disposed, and the EV pellet was rinsed with PBS at 100,000 × g for 70 min at 4 °C. The EV pellet was finally resuspended in PBS and its protein concentration was measured using Bradford Protein Assay Kit (Takara Bio, Shiga, Japan). The obtained EVs were confirmed under transmission electron microscopy (TEM) by the conventional negative staining procedure performed on two different samples. In brief, 10 μl aliquots of EV suspension were sedimented for 2 min onto a 300 mesh, copper/carbon-coated grid and then negatively stained with 2% uranyl acetate and observed with a TEM Hitachi H-7650 at 80 Kv, as previously described^81^. Light-scattering analyses were performed using a Zetasizer Nano-S (Malvern Instruments Ltd., Worcestershire, UK) and a 40-μL cuvette (Malvern Instruments). Samples were equilibrated for 2 min at 25 °C followed by three readings consisting of 10 measurements each. To deplete EVs from tumor cells, cambinol was purchased from Sigma-Aldrich (St. Louis, MO, USA). This was dissolved in dimethylsulfoxide (DMSO, Sigma-Aldrich) according to manufacturer’s instructions. Cambinol dissolved in DMSO was used at 10 μM and the same concentration of DMSO was used for the control. MFS cells were treated with cambinol in serum-free media for 48 hours.

### Isolation of EVs from human serum

EVs were purified from human serum samples by size exclusion chromatography on drip using EV-second (GL sciences, Tokyo, Japan) as previously described^30^. The column was initially equilibrated with 700 μl of PBS twice, followed by a blocking step using 700 μl of FBS. After repeating the wash steps six times with 700 μl of PBS, 200 μl of the collected human serum sample was loaded onto this column followed by collection of 12 consecutive fractions in 100 μl of PBS. CD9 expression in these fractions was measured by CD9 sandwich ELISA and CD9-positive fractions were recognized as the EV-rich portion.

### RNase protection assay

To degrade unprotected RNA and the isolation of RNA only confined within EVs, purified EVs were treated with RNase A (0.5 mg/mL; Takara Bio) for 20 min at 37 °C, followed by enzyme inactivation. Untreated purified EVs were also tested as controls.

### Reverse transcription (RT) and quantitative real-time PCR

Reverse transcription and quantitative real-time PCR were performed, as previously described^30^. The isolated RNAs were reverse transcribed using the TaqMan MicroRNA Reverse Transcription Kit (Applied Biosystems, Foster City, CA, USA). The cDNA products were mixed with 5.0 µL of TaqMan 2× Universal PCR Master mix and 0.50 µL of each primer for qPCR using Agilent Mx3000P (Agilent Technologies, Santa Clara, CA, USA) instrumentation. Data obtained from these procedures were analyzed using the 2^−∆∆Ct^ method^82^. The expression levels of miRNA were normalized using *cel-miR-39* for serum and culture media, and *RNU6B* for tumor and other cells. Differences between the groups are presented as ΔCt, indicating differences between Ct values of miRNAs of interest and Ct values of normalizer miRNAs^30^.

### Western blotting

Western blotting was performed as previously described^30^. Total protein from cells (10 μg) and exosomes (10 μg) was fractionated using an electrophoretic gradient across Mini-PROTEAN tris-glycine extended gels (BIO-RAD, Richmond, CA, USA). Measurement of protein concentration was performed using a Bradford Protein Assay Kit (Takara Bio), according to the manufacturer’s instructions. The gels were then transferred onto Immun-Blot PVDF membranes (BIO-RAD) under wet electrophoretic conditions. The blotted protein was blocked for 1 hr at room temperature with Odyssey blocking buffer in PBS (LI-COR, Lincoln, NE, USA) and was followed by incubation overnight at 4 °C with the following primary antibodies: 1:1000 anti-CD63 mouse monoclonal antibody (BD Biosciences, San Jose, CA, USA); 1:1000 anti-cytochrome-c mouse monoclonal antibody (BD Biosciences); 1:1000 anti-calnexin rabbit monoclonal antibody (Abcam) and 1:1000 hFAB Rhodamine (#12004168 - Bio-Rad). Primary antibodies were detected by IRDye 800CW anti-rabbit IgG and IRDye 680RD anti-mouse IgG secondary antibodies (LI-COR) and were incubated with the protein-blotted membrane for 1 hr at room temperature. Fluorescence was then detected on the Odyssey imaging system (LI-COR).

### Animal experiments

All the animal experiments were approved by the Institutional Animal Care and Use Committee of Okayama University and conducted in compliance with a protocol reviewed by the committee. BALB/c nu/nu female mice were purchased from CLEA Japan Inc. (Tokyo, Japan) at 4 weeks of age. Twelve mice, four mice per group, were inoculated subcutaneously with NMFH-1 cells at a concentration of 5 × 10^6^ cells in 100 μl of RPMI on the right buttock under general anesthesia performed with 2% isoflurane. Four control mice received 100 μl RPMI by the same route. Tumor sizes were measured after one week of injection and then measured once a week. Blood samples were obtained by cardiac puncture at three, five, and seven weeks after injection under general anesthesia. The animals were sacrificed after blood sampling. The blood samples were stored at 4 °C in CAPIJECT micro collection tubes (TERUMO, Tokyo, Japan), and blood processing was done within 2 h of sampling as previously described for patient serum samples.

### miRNA and siRNA transfection

Synthetic miRNA mimics (Mimic *hsa-miR-1260b* and negative control (NC); Gene Design, Ibaraki, Japan) were transfected into Normal Fibroblast (NF) cells at 30 nM using DharmaFECT 1 (Dharmacon, Tokyo, Japan). Twenty-four hours after transfection, NF cells were reseeded. For RNAi experiments, NF cells were transfected with Silencer Select Pre-designed siRNA (Thermo Fisher Scientific) using DharmaFECT 1 (Dharmacon) according to the manufacturer’s protocols.

### Cell invasion assay

NFs (2 × 10^4^ cells) were plated onto 25 μg Matrigel-coated 24-well FluoroBlock cell culture inserts with 8-μm pore sizes (Corning Life Sciences, Durham, NC). Twenty-four hours after NF cells were transfected with *miR-1260b* mimic or miRNA mimic control were cultured, PKH67 (Sigma-Aldrich, St. Louis, MO, USA) -labeled NMFH-1 and NMFH-2 cells in RPMI with 1% bovine serum albumin (Sigma) were incubated on the monolayers of NF cells and allowed to invade for 24 h. Culture medium of the lower chamber consisted of 5% FBS/RPMI. The number of invasive MFS cells was measured by imaging an average of nine random areas per well.

### Identification of miRNA target mRNAs by AGO immunoprecipitation

To confirm the miRNA target mRNA, we performed 3D-Gene Human mRNA Oligo chip 25 k (Toray Industries, Inc., Kamakura, Japan) analysis using samples collected using two experimental methods. First, we extracted total RNA from NF cells transfected with specific miRNA or NC. Second, we extracted RNA using anti-Ago2 antibody immunoprecipitation (Ago2-IP) from NF cells transfected with specific miRNA or NC. We determined the candidate mRNAs that were downregulated with two-fold decreases in the first samples and upregulated with eight-fold increases in the second samples by referring to information in an in-silico database, TargetScan (http://www.targetscan.org).

### Statistical analysis

Statistical differences in the quantified miRNA levels were determined using unpaired *t* test or Analysis of Variance followed by Holm-Sidak’s multiple comparisons test. The Wilcox signed-rank test was used to compare the paired serum samples. Mann-Whitney *U*-test was used for comparison of MFS patients with or without tail-like patterns. In animal experiments, correlations between expression levels of miRNA and tumor sizes were evaluated using Pearson’s correlation coefficient. A two-sided *p*-value of less than 0.05 was considered statistically significant. All statistical analyses were performed using GraphPad Prism v.7.0 (GraphPad Software, San Diego, CA, USA).

### Ethical approval and consent to participate

The study protocol was approved by the Institutional Review Board of the Okayama University. Written informed consent was obtained from all patients and healthy individuals after approval of the study. All the animal experiments were approved by the Institutional Animal Care and Use Committee of Okayama University and carried out in accordance with relevant guidelines and regulations.

### Consent for publication

All the patients involved in our study obtained written consent for publication.

## Supplementary information

Supplementary information.

Supplementary information.

## References

[1] Mentzel T (1996). Myxofibrosarcoma: clinicopathologic analysis of 75 cases with emphasis on the low-grade variant. The American journal of surgical pathology.

[2] Gronchi A (2010). Extremity soft tissue sarcoma in a series of patients treated at a single institution: local control directly impacts survival. Annals of surgery.

[3] Oda Y (2003). Altered expression of cell cycle regulators in myxofibrosarcoma, with special emphasis on their prognostic implications. Human pathology.

[4] Sanfilippo R (2011). Myxofibrosarcoma: prognostic factors and survival in a series of patients treated at a single institution. Ann Surg Oncol.

[5] Iwata S (2014). Impact of infiltrative growth on the outcome of patients with undifferentiated pleomorphic sarcoma and myxofibrosarcoma. Journal of surgical oncology.

[6] Imanishi J (2016). Tail of superficial myxofibrosarcoma and undifferentiated pleomorphic sarcoma after preoperative radiotherapy. Anticancer research.

[7] Kaya M (2008). MRI and histological evaluation of the infiltrative growth pattern of myxofibrosarcoma. Skeletal radiology.

[8] Manoso MW, Pratt J, Healey JH, Boland PJ, Athanasian EA (2006). Infiltrative MRI pattern and incomplete initial surgery compromise local control of myxofibrosarcoma. Clinical orthopaedics and related research.

[9] Lefkowitz RA (2013). Myxofibrosarcoma: prevalence and diagnostic value of the “tail sign” on magnetic resonance imaging. Skeletal radiology.

[10] Huang H-Y, Lal P, Qin J, Brennan MF, Antonescu CR (2004). Low-grade myxofibrosarcoma: a clinicopathologic analysis of 49 cases treated at a single institution with simultaneous assessment of the efficacy of 3-tier and 4-tier grading systems. Human pathology.

[11] Waters B (2007). Low-grade myxofibrosarcoma: CT and MRI patterns in recurrent disease. American Journal of Roentgenology.

[12] Fernebro, J. *et al*. Focus on the tumour periphery in MRI evaluation of soft tissue sarcoma: infiltrative growth signifies poor prognosis. *Sarcoma* **2006** (2006). 10.1155/SRCM/2006/21251 PMC1779504 17496992

[13] Manoso MW (2006). Infiltrative MRI pattern and incomplete initial surgery compromise local control of myxofibrosarcoma. Clinical orthopaedics and related research.

[14] Yoo HJ (2014). MR imaging of myxofibrosarcoma and undifferentiated sarcoma with emphasis on tail sign; diagnostic and prognostic value. European radiology.

[15] Willems SM, Debiec-Rychter M, Szuhai K, Hogendoorn PC, Sciot R (2006). Local recurrence of myxofibrosarcoma is associated with increase in tumour grade and cytogenetic aberrations, suggesting a multistep tumour progression model. Modern Pathology.

[16] Calin GA (2005). A MicroRNA signature associated with prognosis and progression in chronic lymphocytic leukemia. The New England journal of medicine.

[17] Calin GA, Croce CM (2006). MicroRNA signatures in human cancers. Nature reviews. Cancer.

[18] Esquela-Kerscher A, Slack FJ (2006). Oncomirs - microRNAs with a role in cancer. Nature reviews. Cancer.

[19] Ambros V (2004). The functions of animal microRNAs. Nature.

[20] Bartel DP (2004). MicroRNAs: genomics, biogenesis, mechanism, and function. Cell.

[21] Fujiwara T (2014). Clinical relevance and therapeutic significance of microRNA‐133a expression profiles and functions in malignant osteosarcoma‐initiating cells. Stem Cells.

[22] Kosaka N, Iguchi H, Ochiya T (2010). Circulating microRNA in body fluid: a new potential biomarker for cancer diagnosis and prognosis. Cancer science.

[23] Valadi H (2007). Exosome-mediated transfer of mRNAs and microRNAs is a novel mechanism of genetic exchange between cells. Nature cell biology.

[24] Kosaka N, Iguchi H, Ochiya T (2010). Circulating microRNA in body fluid: a new potential biomarker for cancer diagnosis and prognosis. Cancer Sci.

[25] Taylor DD, Gercel-Taylor C (2008). MicroRNA signatures of tumor-derived exosomes as diagnostic biomarkers of ovarian cancer. Gynecologic oncology.

[26] Ogawa Y, Kanai-Azuma M, Akimoto Y, Kawakami H, Yanoshita R (2008). Exosome-like vesicles with dipeptidyl peptidase IV in human saliva. Biological and Pharmaceutical Bulletin.

[27] Cortez MA (2011). MicroRNAs in body fluids—the mix of hormones and biomarkers. Nature reviews Clinical oncology.

[28] Simpson RJ, Lim JW, Moritz RL, Mathivanan S (2009). Exosomes: proteomic insights and diagnostic potential. Expert review of proteomics.

[29] Fujiwara T (2014). Clinical relevance and therapeutic significance of microRNA-133a expression profiles and functions in malignant osteosarcoma-initiating cells. Stem Cells.

[30] Uotani K (2017). Circulating MicroRNA-92b-3p as a novel biomarker for monitoring of synovial sarcoma. Scientific reports.

[31] Valadi H (2007). Exosome-mediated transfer of mRNAs and microRNAs is a novel mechanism of genetic exchange between cells. Nat Cell Biol.

[32] Van Niel G, D’Angelo G, Raposo G (2018). Shedding light on the cell biology of extracellular vesicles. Nature reviews Molecular cell biology.

[33] Al-Nedawi K (2008). Intercellular transfer of the oncogenic receptor EGFRvIII by microvesicles derived from tumour cells. Nat Cell Biol.

[34] Grange C (2011). Microvesicles released from human renal cancer stem cells stimulate angiogenesis and formation of lung premetastatic niche. Cancer research.

[35] Lima LG, Chammas R, Monteiro RQ, Moreira ME, Barcinski MA (2009). Tumor-derived microvesicles modulate the establishment of metastatic melanoma in a phosphatidylserine-dependent manner. Cancer Lett.

[36] Webber J, Steadman R, Mason MD, Tabi Z, Clayton A (2010). Cancer exosomes trigger fibroblast to myofibroblast differentiation. Cancer research.

[37] Zhou W (2014). Cancer-secreted miR-105 destroys vascular endothelial barriers to promote metastasis. Cancer cell.

[38] Figuera-Losada, M. *et al*. Cambinol, a novel inhibitor of neutral sphingomyelinase 2 shows neuroprotective properties. *PloS one* **10** (2015). 10.1371/journal.pone.0124481 PMC4444023 26010541

[39] Menck K (2017). Neutral sphingomyelinases control extracellular vesicles budding from the plasma membrane. Journal of extracellular vesicles.

[40] Chen, X. *et al*. Characterization of microRNAs in serum: a novel class of biomarkers for diagnosis of cancer and other diseases. *Cell research* **18** (2008). 10.1038/cr.2008.282 18766170

[41] Chen X (2012). Identification of ten serum microRNAs from a genome‐wide serum microRNA expression profile as novel noninvasive biomarkers for nonsmall cell lung cancer diagnosis. International journal of cancer.

[42] Abd-El-Fattah AA, Sadik NAH, Shaker OG, Aboulftouh ML (2013). Differential microRNAs expression in serum of patients with lung cancer, pulmonary tuberculosis, and pneumonia. Cell biochemistry and biophysics.

[43] Zhu W, Qin W, Atasoy U, Sauter ER (2009). Circulating microRNAs in breast cancer and healthy subjects. BMC research notes.

[44] Roth C (2010). Circulating microRNAs as blood-based markers for patients with primary and metastatic breast cancer. Breast Cancer Research.

[45] Sun Y (2012). Serum microRNA-155 as a potential biomarker to track disease in breast cancer. PloS one.

[46] Li, A. *et al*. MicroRNA array analysis finds elevated serum miR-1290 accurately distinguishes patients with low-stage pancreatic cancer from healthy and disease controls. *Clinical cancer research* (2013). 10.1158/1078-0432.CCR-12-3092 PMC3707520 23697990

[47] Mitchell PS (2008). Circulating microRNAs as stable blood-based markers for cancer detection. Proceedings of the National Academy of Sciences.

[48] Ogata-Kawata H (2014). Circulating exosomal microRNAs as biomarkers of colon cancer. PLoS One.

[49] Ng, E. K. *et al*. Differential expression of microRNAs in plasma of colorectal cancer patients: a potential marker for colorectal cancer screening. *Gut* (2009). 10.1136/gut.2008.167817 19201770

[50] Tsujiura M (2010). Circulating microRNAs in plasma of patients with gastric cancers. British journal of cancer.

[51] Zhu C (2014). A five-microRNA panel in plasma was identified as potential biomarker for early detection of gastric cancer. British journal of cancer.

[52] Li Z (2015). By downregulating TIAM1 expression, microRNA-329 suppresses gastric cancer invasion and growth. Oncotarget.

[53] Qi P (2011). Serum microRNAs as biomarkers for hepatocellular carcinoma in Chinese patients with chronic hepatitis B virus infection. PloS one.

[54] Shen J (2013). Exploration of genome-wide circulating microRNA in hepatocellular carcinoma: MiR-483-5p as a potential biomarker. Cancer Epidemiology and Prevention Biomarkers.

[55] Li L-M (2010). Serum microRNA profiles serve as novel biomarkers for HBV infection and diagnosis of HBV-positive hepatocarcinoma. Cancer research.

[56] Fricke A (2015). Identification of a blood-borne miRNA signature of synovial sarcoma. Molecular cancer.

[57] Miyachi M (2010). Circulating muscle-specific microRNA, miR-206, as a potential diagnostic marker for rhabdomyosarcoma. Biochemical and biophysical research communications.

[58] Weng Y, Chen Y, Chen J, Liu Y, Bao T (2013). Identification of serum microRNAs in genome-wide serum microRNA expression profiles as novel noninvasive biomarkers for malignant peripheral nerve sheath tumor diagnosis. Med Oncol.

[59] Pigati L (2010). Selective release of microRNA species from normal and malignant mammary epithelial cells. PloS one.

[60] Chan M (2013). Identification of circulating microRNA signatures for breast cancer detection. Clinical cancer research, clincanres..

[61] Wang K, Zhang S, Weber J, Baxter D, Galas DJ (2010). Export of microRNAs and microRNA-protective protein by mammalian cells. Nucleic acids research.

[62] Hirata H (2014). Genistein downregulates onco-miR-1260b and upregulates sFRP1 and Smad4 via demethylation and histone modification in prostate cancer cells. British journal of cancer.

[63] Hirata H (2013). Genistein downregulates onco-miR-1260b and inhibits Wnt-signalling in renal cancer cells. British journal of cancer.

[64] Xu L (2015). Overexpression of miR-1260b in non-small cell lung cancer is associated with lymph node metastasis. Aging and disease.

[65] Liu D-R, Guan Q-L, Gao M-T, Jiang L, Kang H-X (2016). miR-1260b is a potential prognostic biomarker in colorectal cancer. Medical science monitor: international medical journal of experimental and clinical research.

[66] Tominaga, N. *et al*. Brain metastatic cancer cells release microRNA-181c-containing extracellular vesicles capable of destructing blood–brain barrier. *Nature communications* **6** (2015). 10.1038/ncomms7716 PMC4396394 25828099

[67] Zhang Y (2010). Secreted monocytic miR-150 enhances targeted endothelial cell migration. Molecular cell.

[68] Yang M (2011). Microvesicles secreted by macrophages shuttle invasion-potentiating microRNAs into breast cancer cells. Molecular cancer.

[69] Liao J, Liu R, Shi Y-J, Yin L-H, Pu Y-P (2016). Exosome-shuttling microRNA-21 promotes cell migration and invasion-targeting PDCD4 in esophageal cancer. International journal of oncology.

[70] Nakamura K (2017). Exosomes Promote Ovarian Cancer Cell Invasion through Transfer of CD44 to Peritoneal Mesothelial Cells. Mol Cancer Res.

[71] Sakha, S., Muramatsu, T., Ueda, K. & Inazawa, J. Exosomal microRNA miR-1246 induces cell motility and invasion through the regulation of DENND2D in oral squamous cell carcinoma. *Scientific Reports* **6** (2016). 10.1038/srep38750 PMC5144099 27929118

[72] Kahlert C, Kalluri R (2013). Exosomes in tumor microenvironment influence cancer progression and metastasis. Journal of molecular medicine.

[73] Zhuang G (2012). Tumour‐secreted miR‐9 promotes endothelial cell migration and angiogenesis by activating the JAK‐STAT pathway. The EMBO journal.

[74] Frank M, Kemler R (2002). Protocadherins. Current opinion in cell biology.

[75] Wang C (2012). Downregulation of PCDH9 predicts prognosis for patients with glioma. Journal of Clinical Neuroscience.

[76] Tayrac MD (2009). Integrative genome‐wide analysis reveals a robust genomic glioblastoma signature associated with copy number driving changes in gene expression. Genes, Chromosomes and Cancer.

[77] Zhu P (2014). Protocadherin 9 inhibits epithelial–mesenchymal transition and cell migration through activating GSK-3β in hepatocellular carcinoma. Biochemical and biophysical research communications.

[78] Hur K (2017). Circulating microRNA-203 predicts prognosis and metastasis in human colorectal cancer. Gut.

[79] Kikuta K (2015). An analysis of factors related to the tail-like pattern of myxofibrosarcoma seen on MRI. Skeletal radiology.

[80] Katano H, Nishikawa Y, Yamada H, Yamada K, Mase M (2018). Differential expression of microRNAs in severely calcified carotid plaques. Journal of Stroke and Cerebrovascular Diseases.

[81] Jin Y (2016). DNA in serum extracellular vesicles is stable under different storage conditions. BMC cancer.

[82] Livak KJ, Schmittgen TD (2001). Analysis of relative gene expression data using real-time quantitative PCR and the 2− ΔΔCT method. methods.

