# Supplementary material for: Clinical relevance and functional significance of cell-free microRNA-1260b expression profiles in infiltrative myxofibrosarcoma
Source: Sci Rep. 2020 Jun 10;10:9414. doi: 10.1038/s41598-020-66120-8 (PMC7287053; doi:10.1038/s41598-020-66120-8)
Supplement: Supplementary file 1 — Supplementary information. [file 41598_2020_66120_MOESM1_ESM.pdf]

Supplementary information

Clinical relevance and functional significance of cell-free microRNA-1260b expression profiles in infiltrative myxofibrosarcoma

Takuya Morita,[1] Tomohiro Fujiwara,[1,2] Koji Uotani,[1] Aki Yoshida,[1] Masahiro Kiyono,[1] Suguru Yokoo,[1] Joe Hasei,[1] Toshiyuki Kunisada,[1] Toshifumi Ozaki[1]

[1.] Department of Orthopedic Surgery, Okayama University Graduate School of Medicine, Dentistry, and Pharmaceutical Sciences, Okayama, Japan

[2.] Department of Orthopaedic Oncology, Memorial Sloan-Kettering Cancer Centre, New York, USA

Running title:

Clinical and functional significance of circulating *miR-1260b* in myxofibrosarcoma

Supplementary Table 1. Clinical characteristics of patients with myxofibrosarcoma who were included in the array analyses.

No.	Gender	Age	Site	Depth	MRI pattern	Size (cm^3)	FNCLCC grade	Local recurrence	Metastasis	Disease status
1	M	60	Extremity	Deep	Tail-like	24.8	2	+	+	DOD
2	F	90	Extremity	Superficial	Solid	32	2	-	-	CDF
3	F	70	Extremity	Superficial	Tail-like	8.3	2	-	-	CDF
4	M	78	Extremity	Deep	Tail-like	144.5	2	-	-	CDF
5	M	75	Trunk	Superficial	Tail-like	40.5	2	-	-	CDF

Supplementary Table 2. Characteristics of non-sarcoma patients who were included in the array analyses.

No.	Gender	Age	Diagnosis
1	M	64	Lipoma
2	F	65	Lipoma
3	F	71	GCTTS
4	M	83	GCTTS
5	F	69	Schwannoma

Abbreviation: GCTTS: Giant cell tumor of tendon sheath

Supplementary Table 3. Characteristics of healthy individuals who were included in the array analyses.

No.	Gender	Age	Disease-state
1	F	56	Healthy individual
2	F	58	Healthy individual
3	F	54	Healthy individual
4	F	34	Healthy individual
5	F	33	Healthy individual
6	F	42	Healthy individual
7	F	62	Healthy individual
8	F	64	Healthy individual
9	M	32	Healthy individual

Supplementary Table 4. Differentially expressed miRNAs in MFS patients vs age-matched patients with benign tumor patients (NON; $p<0.005$).

See Supplementary Data 2 File

Supplementary Table 5. Differentially expressed miRNAs in MFS patients vs Healthy individuals (CONT; $p<0.005$)

See Supplementary Data 2 File

Supplementary Table 6. Differentially expressed miRNAs in MFS patients vs Healthy individuals or benign tumor patients

**miRNA**	***P* value**		**Fold change**	
	MFS vs CONT	MFS vs NON	MFS vs CONT	MFS vs NON
miR-642a	5.3E-02	8.8E-02	3.0	2.1
miR-1260b	5.4E-03	3.6E-03	5.1	2.3
miR-4286	1.6E-03	5.63.E-02	4.1	2.7
miR-4313	4.4E-03	6.6E-02	4.1	1.7

Supplementary Table 7. Downregulated genes determined by a combination of Input-IP after transfection with miR-1260b compared to miR-NC in NFs (< 2-fold decrease).

See Supplementary Data 2 File

Supplementary Table 8. Upregulated genes determined by a combination of AGO2-IP after transfection with miR-1260b compared to miR-NC in NFs (> 4-fold increase).

See Supplementary Data 2 File

Supplementary Table 9. Candidates of *miR-1260b* target genes (selected from Supplementary Table 7 and Supplementary Table 8).

Ensembl_ID	Symbol	Description	Global normalization			
			AGO2-1260b	AGO2-NC	Input-1260b	Input-NC
ENSG00000256683	ZNF350	zinc finger protein 350	3174	125	110	493
ENSG00000067066	SP100	SP100 nuclear antigen	1682	337	57	297
ENSG00000172046	USP19	ubiquitin specific peptidase 19	996	98	21	46
ENSG00000167384	ZNF180	zinc finger protein 180	565	27	10	22
ENSG00000186300	ZNF555	zinc finger protein 555	465	10	6	29
ENSG00000169957	ZNF768	zinc finger protein 768	431	80	5	11
ENSG00000182175	RGMA	repulsive guidance molecule family member a	317	29	7	17
ENSG00000158805	ZNF276	zinc finger protein 276	270	6	5	24
ENSG00000184226	PCDH9	protocadherin 9	222	27	13	27
ENSG00000004700	RECQL	RecQ like helicase	196	9	44	95
ENSG00000235109	ZSCAN31	zinc finger and SCAN domain containing 31	181	25	14	32
ENSG00000121274	PAPD5	PAP associated domain containing 5	174	24	10	39
ENSG00000083817	ZNF416	zinc finger protein 416	146	27	17	42
ENSG00000076641	PAG1	phosphoprotein membrane anchor with glycosphingolipid microdomains 1	106	14	23	49
ENSG00000165280	VCP	valosin containing protein	102	24	22	45

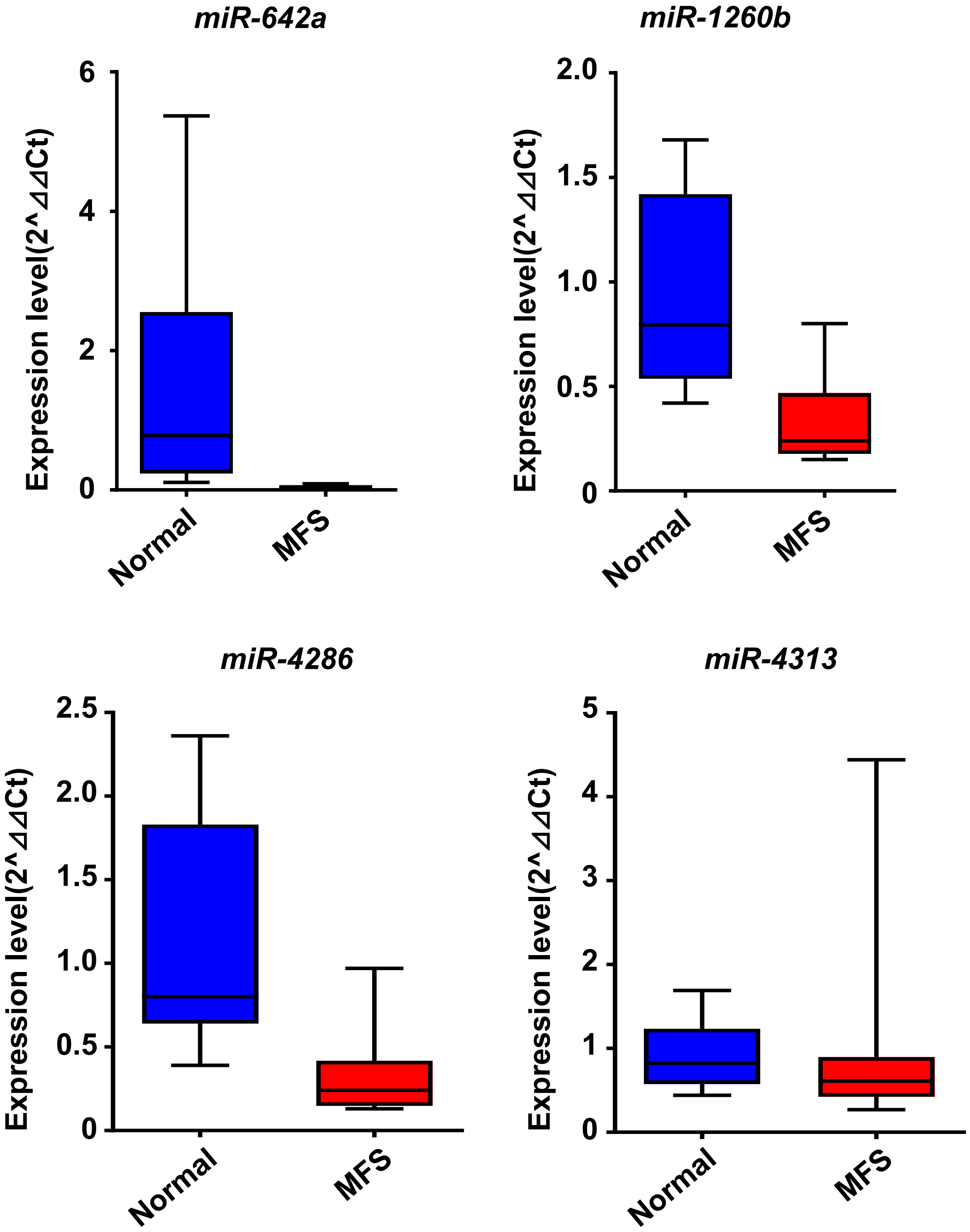

Supplementary Figure 1. The miRNA expression levels in MFS tumor tissues and controls (normal muscle). All of these four candidates (*miR-642a, miR-1260b, miR-4286 and miR-4313*) were not significantly upregulated in MFS tissue specimens compared to the normal tissue specimens.

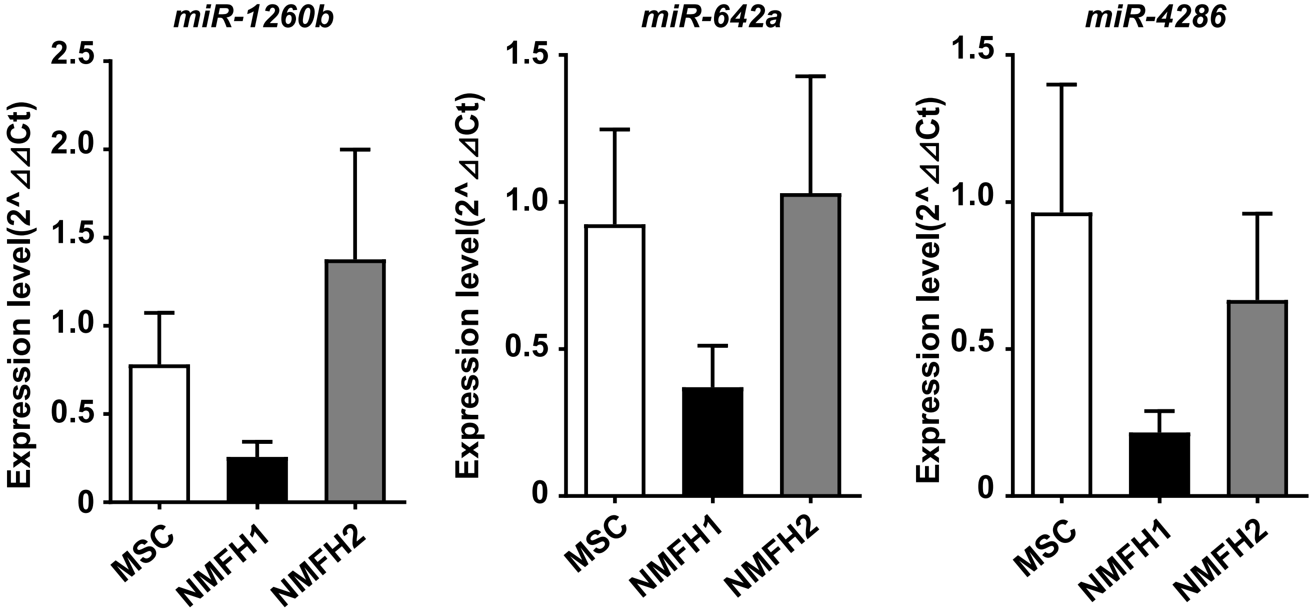

Supplementary Figure 2. The cellular miRNA expression levels in MFS cells and hMSCs. The expression levels of *miR-1260b, miR-642a*, and *miR-4286* were not significantly upregulated in MFS cells compared to the hMSCs.

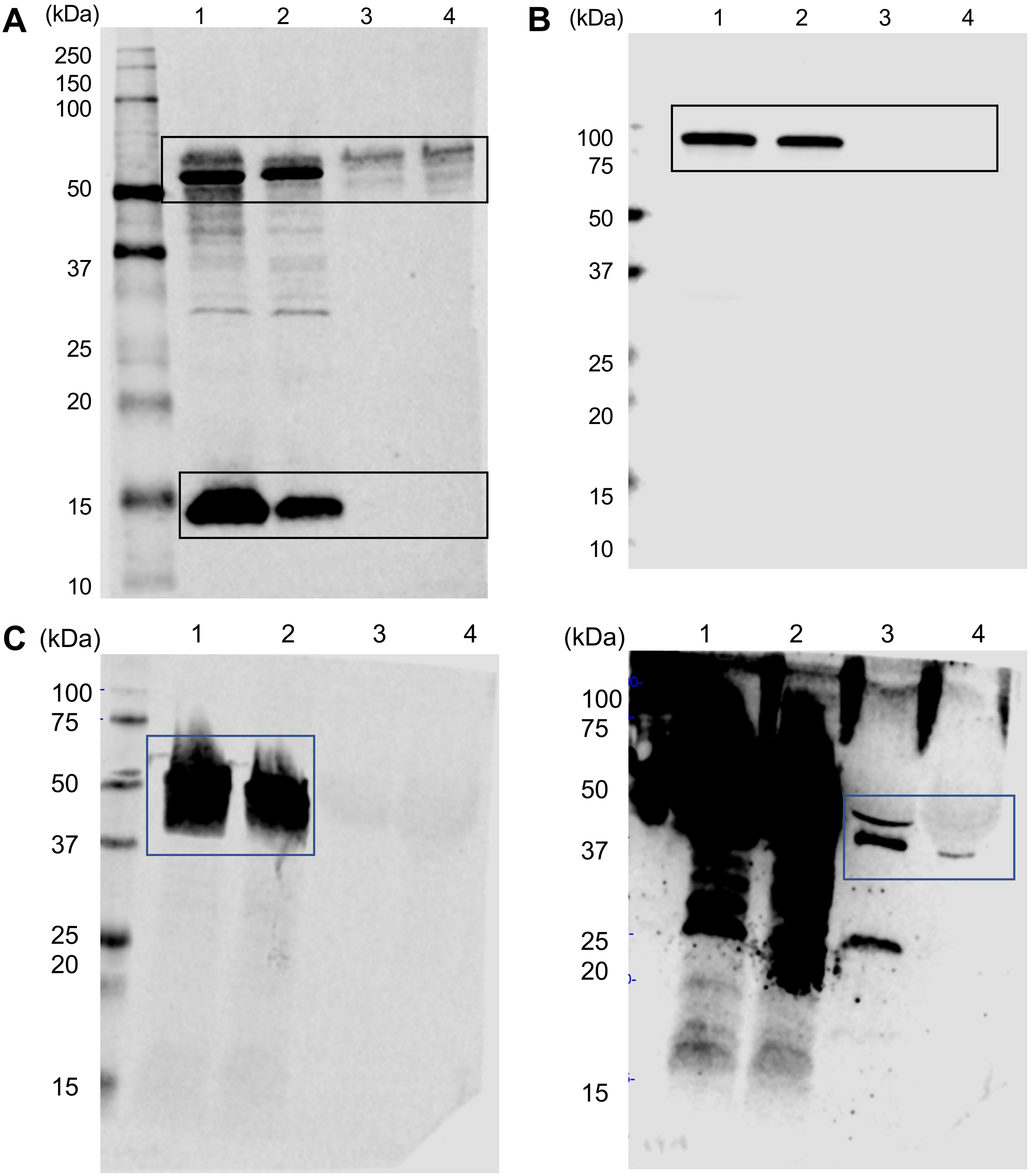

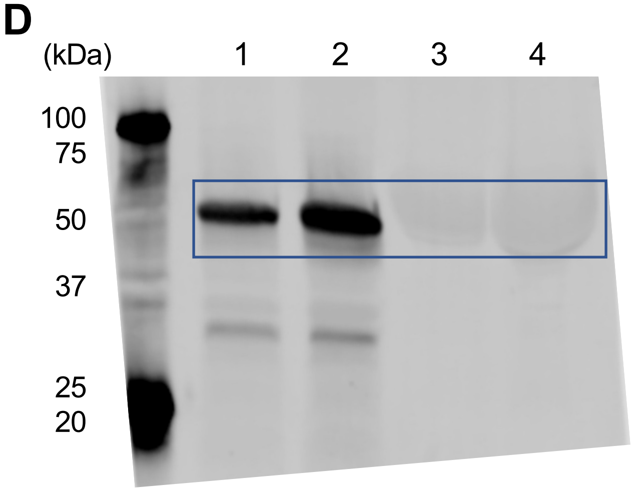

Lane No.	Type of samples	Cell lines
1	Cells	NMHF1
2	Cells	NMFH2
3	EVs	NMHF1
4	EVs	NMFH2

Supplementary Figure 3. Full-length blots shown in Figure 3C. (A) CD63 (53kDa) and cytochrome-C (15kDa). (B) Calnexin (90kDa). (C) Vimentin (54kDa). (D)Tublin (50kDa).

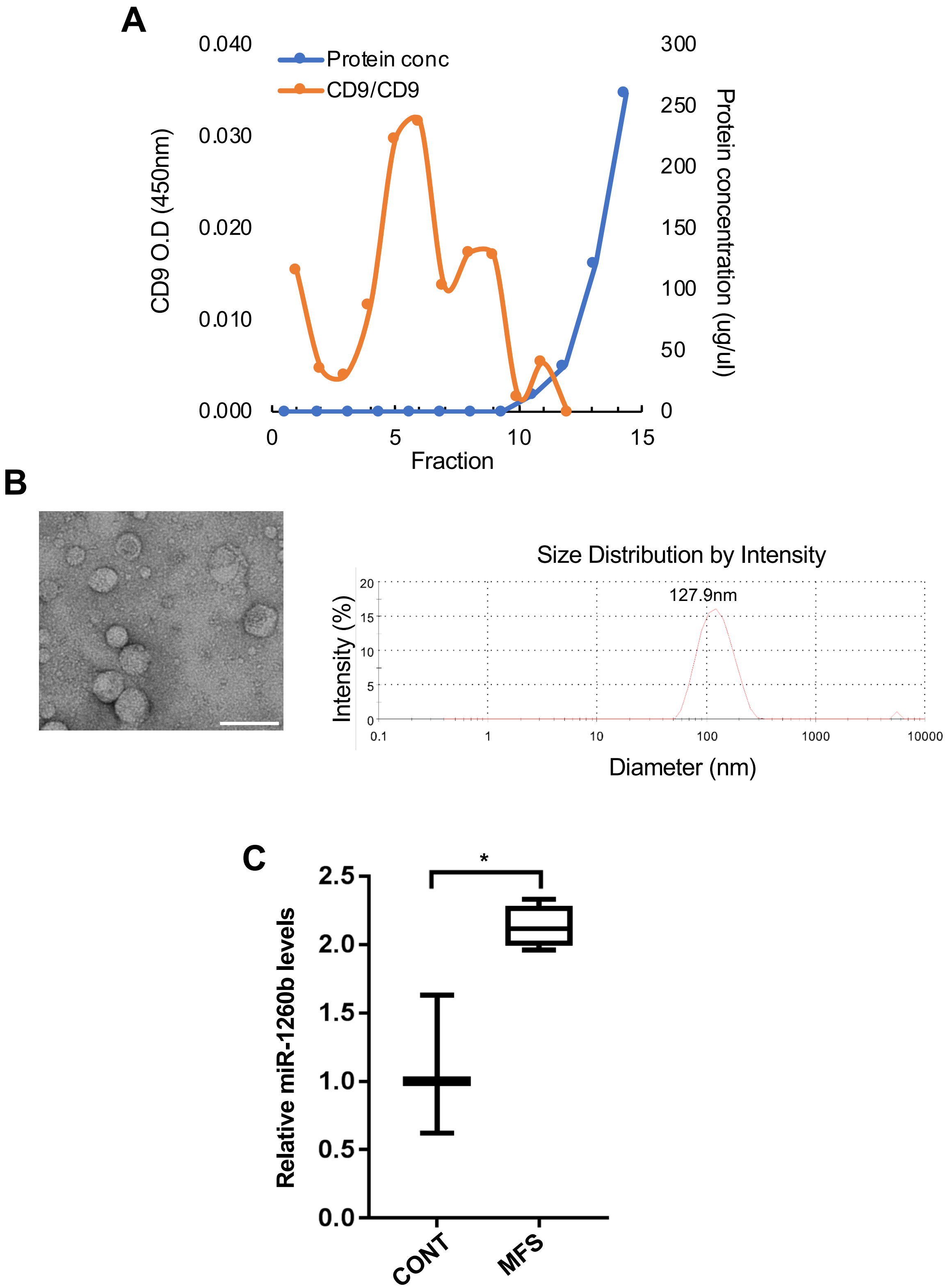

Supplementary Figure 4. *miR-1260b* expression levels in the EVs from human serum. (A) Isolation of EVs using EV-Second method. CD9-positive fractions (5-7 fractions) were recognized as the EV-rich portion. (B) The collected EVs from fraction 5 confirmed by TEM (left) and ZetaSizer (right) (C) *miR-1260b* expression levels in EVs from MFS patients (MFS) and healthy individuals (CONT). *$p < 0.05$.

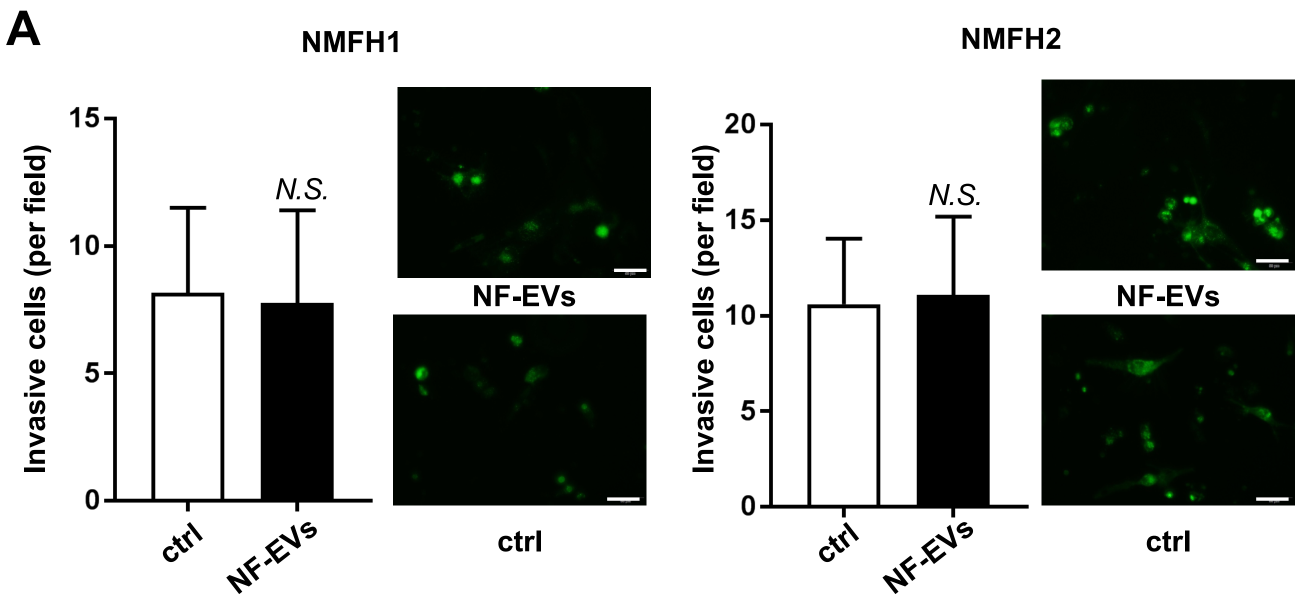

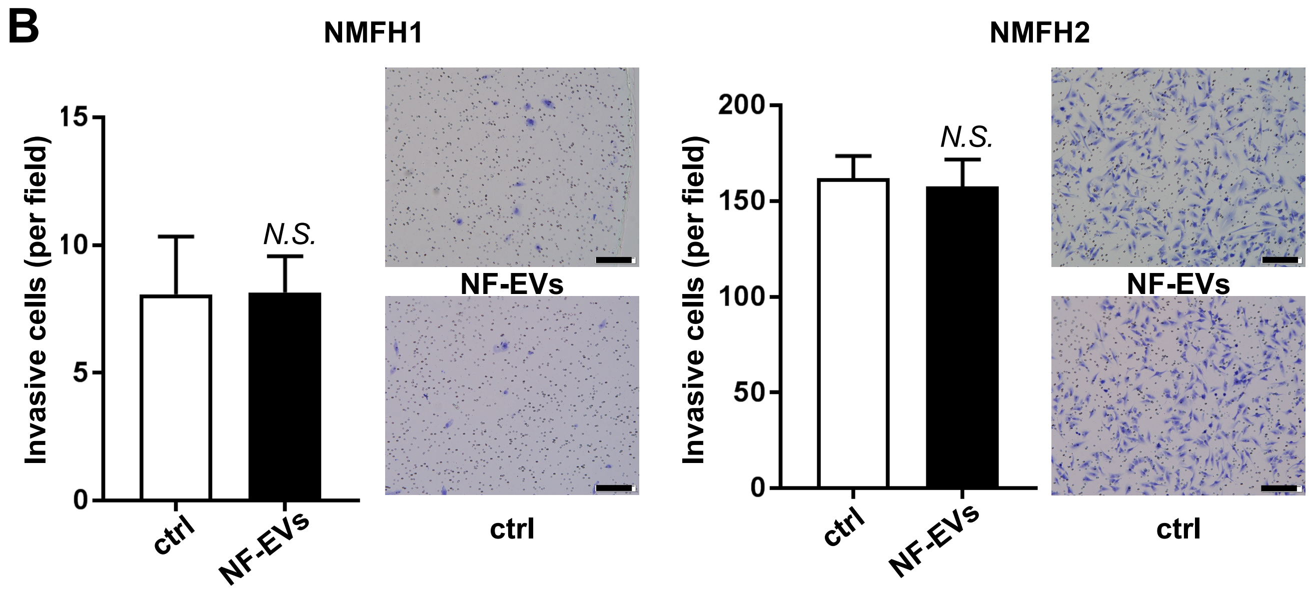

Supplementary Figure 5. Invasion assay. (A) NMFH1 and NMFH2 cells educated by NF cells-derived EVs and control PBS did not show significant difference in cellular invasion. Data are mean ± S.D. (n = 3 in each group) Scale bar = 50 µm. (B) NF cells-derived EVs educated NMFH1 and NMFH2 cells did not stimulate cellular invasion of MFS cells. Left, NMFH-1; right, NMFH-2. Data are mean ± S.D. (n = 3 in each group) Scale bar = 200 µm. *N.S.*, not significant; Student's *t* test.
